# The auditory outcomes in non-blast related traumatic brain injury and the role of severity, aetiology and gender: a scoping review

**DOI:** 10.3389/fneur.2025.1589117

**Published:** 2025-07-10

**Authors:** Kübra Bölükbaş, Laura Edwards, Olivia R. Phillips, Kathryn Fackrell

**Affiliations:** ^1^Hearing Sciences, Division of Mental Health and Clinical Neuroscience, School of Medicine, University of Nottingham, Nottingham, United Kingdom; ^2^National Institute of Health and Social Research (NIHR) Nottingham Biomedical Research Centre, Nottingham, United Kingdom; ^3^Division of Rehabilitation Medicine, University Hospitals of Derby and Burton NHS Foundation Trust, Derby, United Kingdom; ^4^Centre for Rehabilitation and Ageing Research, School of Medicine, University of Nottingham, Nottingham, United Kingdom; ^5^Lifespan and Population Health, School of Medicine, University of Nottingham, Nottingham, United Kingdom

**Keywords:** traumatic brain injury, auditory, hearing loss, tinnitus, hyperacusis, TBI severity, aetiology, gender

## Abstract

**Introduction:**

Traumatic brain injury (TBI) can cause a wide range of auditory outcomes. This review aimed to investigate common auditory outcomes associated with TBI and explore variations based on severity, aetiology, and gender.

**Methods:**

A scoping review was conducted using an established methodological framework, which involved electronic and manual searches of databases and journals. Records published in English were included, which focused on auditory outcomes and assessments associated with non-blast related TBI in individuals 18 years and older. From 19,031 records, 61 met the inclusion criteria. Data were collated and categorized based on the study objectives.

**Results:**

Pure-tone audiometry (56/61) was the most commonly used hearing assessment, followed by otoscopy (27/61), whilst for tinnitus and hyperacusis assessments varied from questionnaires to self-reported problems. Different types of hearing loss were reported; conductive to mixed, of these 41% noted sensorineural hearing loss (SNHL). Normal hearing (≤ 20/25 dB HL) was reported in 31% (19/61) of the studies, however, five studies found abnormal results in central auditory tests despite normal hearing. Severe TBI was reported more frequently compared to other severities (10/23). Although SNHL was noted in 4 studies related to severe TBI, various outcomes were observed ranging from normal hearing to total deafness. Motor-vehicle accidents (MVA) were the most common aetiology (36/61), followed by falls, assaults, and sports injuries. Following MVA, SNHL was observed in 12 studies and CHL was observed across 10 studies. Out of 61 articles, 53% included only male patients, and SNHL was observed more frequently in males (17/33), whilst normal hearing and other types of hearing loss were noted in both genders.

**Conclusion:**

TBI-related auditory impairments are complex, with inconsistent assessment methods and reporting gaps complicating data synthesis. Standardized clinical practices and screening guidelines are crucial for improving auditory assessment and management in this population.

## 1 Introduction

Traumatic brain injury (TBI), specified as a traumatic structural injury and/or physiological deterioration of brain functions caused by an external force (1), can result in many physical, cognitive, behavioral and emotional impairments (2–4). TBI is estimated to affect 64–74 million people worldwide each year (5). There are different ways of classifying the severity of TBI; most commonly, the Glasgow Coma Scale (GCS) at the time of injury and duration of post-traumatic amnesia are used to classify TBI as mild, moderate or severe (6). The most common type of TBI is “mild” (GCS 13–15; post-traumatic amnesia duration <24 h; loss of consciousness <30 min) with males aged 18–65 years being at highest risk of experiencing TBI (7). There is a range of causes associated with TBI, including falls, traffic accidents, assaults, sports injuries (non-blast related) and explosions (blast-related). While there will be some similarities in the way the brain is affected by each aetiology, blast-related TBI has consistently been recognized to have some particular mechanisms- e.g., typically involves the transmission of high-pressure waves through air and/or fluid-filled spaces, which can cause widespread damage to the brain and inner ear by disrupting vascular structures, neuronal tissue, and the blood–brain barrier (8, 9). In contrast, non-blast related TBI generally results from mechanical forces such as direct impact or acceleration-deceleration forces, and may lead to more focal injuries including contusions, diffuse axonal injury and blood–brain barrier disruption (10). While both mechanisms can affect similar structures, the pattern and distribution of the resulting injuries may differ. Blast-related TBI is also more likely to be seen in a military population, which may differ from the civilian population in a range of characteristics. Given the various injury patterns and population characteristics, this review focuses specifically on non-blast related TBI, as it more accurately reflects the injuries encountered in civilian life and may offer a clearer framework for understanding auditory outcomes.

Auditory conditions (such as hearing loss, tinnitus (ringing in the ear), hyperacusis (sound sensitivity)) can be observed in patients with TBI due to impairments or damage to the central and peripheral auditory systems (11, 12). Characteristically, auditory conditions occur directly in fractures or damages in the temporal bone region. For instance, sensorineural hearing loss (SNHL) is in general associated with transverse fractures, whilst conductive hearing loss (CHL) is associated with longitudinal fractures (13). In a nationwide population-based study in Taiwan, individuals with TBI were found to have a 2.125 times higher risk of developing hearing loss (14). Moreover, in a study investigating trauma-related tinnitus, 1.7% of 1,604 patients reporting experiencing tinnitus due to head trauma (12).

Although there are studies assessing auditory functions related to TBI, there is currently no comprehensive review synthesising common auditory findings related to non-blast related TBI, in particular aetiology and severity of TBI related to auditory conditions. Addressing this gap in the knowledge will provide evidence to clarify the diagnosis and treatment methods, to help establish appropriate management strategies for auditory conditions in this patient group, and in turn reduce the negative effects of these comorbidities caused by TBI.

Specifically, the objectives here are to identify:

- What are the common auditory impairments of non-blast related TBI,
- Whether auditory outcomes vary according to severity of non-blast related TBI,
- Whether auditory outcomes vary according to aetiology of non-blast related TBI,
- Whether auditory outcomes vary by gender following non-blast related TBI.
- For this purpose, a scoping review was determined to be the most appropriate method, as it is specifically designed to explore broad and diverse research questions, map the literature, summarize the findings, and synthesize the evidence obtained from a range of study designs (15, 16).

## 2 Materials and methods

The methodology of this scoping review was conducted in accordance with the 6-stages framework developed by Arksey and O’Malley (15): (1) identifying the research question(s), (2) identifying relevant studies using appropriate keywords, (3) selecting relevant studies through iterative scanning of titles, abstracts, and full-texts, (4) extraction and charting the data, (5) collating, summarising and reporting of the results, (6) clinician review. The review is reported following the PRISMA-S guidelines (17) (see Supplementary Appendix Table 1).

### 2.1 Identifying the research question(s)

For this purpose, research questions (listed above) were developed in consensus with the team members based on existing knowledge of the field and literature.

### 2.2 Identifying relevant studies

#### 2.2.1 Eligibility criteria

Records were included if they reported studies/cases in which adults (≥18 years old) reported experiencing non-blast related TBI with associated hearing impairments, and hearing outcomes and assessment were reported (including self-reported auditory outcomes). Records were eligible if they reported symptoms or assessments pre-treatment and originated from cohort studies, case series, and case studies, as well as grey literature sources, particularly dissertations and theses. All included records were published in the English language and have full-text. Cases that did not meet our inclusion criteria were removed from the case series studies.

Records were excluded if the studies were reporting adults who may have experienced blast-related TBI, TBI in childhood, whiplash injuries, or non-TBI conditions (e.g., strokes, acoustic neuroma) or they did not clearly define TBI or provide evidence of structural injury or functional deterioration due to TBI. Records involving participants with pre-existing audiological impairments before the TBI, where the aetiology of TBI was not reported, and/or records whose primary aim was to determine the reliability and validity of tests were excluded. Review articles (including systematic reviews), book chapters, randomized control trials, qualitative research studies and any sources reporting personal/expert opinions were excluded.

#### 2.2.2 Search strategy

The research strategy was developed by the research team and was supported by a medical information specialist (Dr Farhad Shokraneh). The search was conducted following Cochrane Handbook (18) and Cochrane’s MECIR (19) and PRESS guideline for peer-reviewing the search strategies (20). Electronic databases were searched including Embase, MEDLINE, ProQuest Dissertations & Theses A&I, PsycINFO, Science Citation Index Expanded and SPORTDiscus in May 2022. The search strategy included keywords on TBI, auditory and vestibular conditions (a separate review was conducted for vestibular outcomes). These were reviewed and revised following a primary search (see Supplementary Appendix Table 2 for search strategy). Specific search term strategies were applied in each search engine, searching article topics, titles, abstracts, and keywords. Filters were applied to retrieve articles in the English language and human participant studies only, when possible. There was no restriction in the search period. To seek further eligible documents for inclusion, manual searches of the reference lists and most common journals (determined using the interquartile rule for outliers) in which eligible records had been sourced were conducted. The final database and manual searches were conducted in September 2024.

### 2.3 Study selection

Records identified through electronic databases were exported with citation, title and abstract into EndNote (version X9), where duplicates were removed, before records were imported into Rayyan (21) for screening. Records were independently screened by four researchers (KB, OP, LE, KF), starting with the title and abstract, before moving onto the full text. Lead researcher (KB) screened all records. The records obtained as a result of the manual search were subjected to full-text screening. When disagreements arose regarding the inclusion or exclusion of any given record, the reviewers discussed their reasons until agreement was reached or a third reviewer was consulted to reach a majority decision.

### 2.4 Extraction and charting of the data

A data extraction form was developed in Microsoft Excel and piloted on five included records and was subsequently modified following team discussions. Data from each record were extracted by lead researcher (KB) and checked by KF. Data were extracted on study characteristics, study population, TBI characteristics, audiological complaints and assessments/outcomes, and limitations (Box 1).

Box 1 **Data extraction fields**

Authors

Year of publication

Country where study was conducted

Study Title

Aim of Study

Study Design

Study Population

Sample Size

Age

Gender

Classification method for TBI

Severity of TBI

Causes/ateiology of TBI

Status pre/post-TBI

Presence of coma

Radiological results

List of auditory complaints

List of audiological assesment tools

Audiological outcomes

Assesment time since injury

Single or repeated assessments

Study limitations

### 2.5 Collating, summarising and reporting results

Extracted data were collated and categorized based on the objectives of our research. Similar findings were grouped into categories such as auditory outcomes, severity of TBI, aetiology, and gender effects. Data were then summarized to identify common patterns and significant variations in auditory outcomes.

### 2.6 Clinician review

After the categories were identified, categorized outcomes were also examined by clinician LE.

## 3 Results

**Figure 1:**
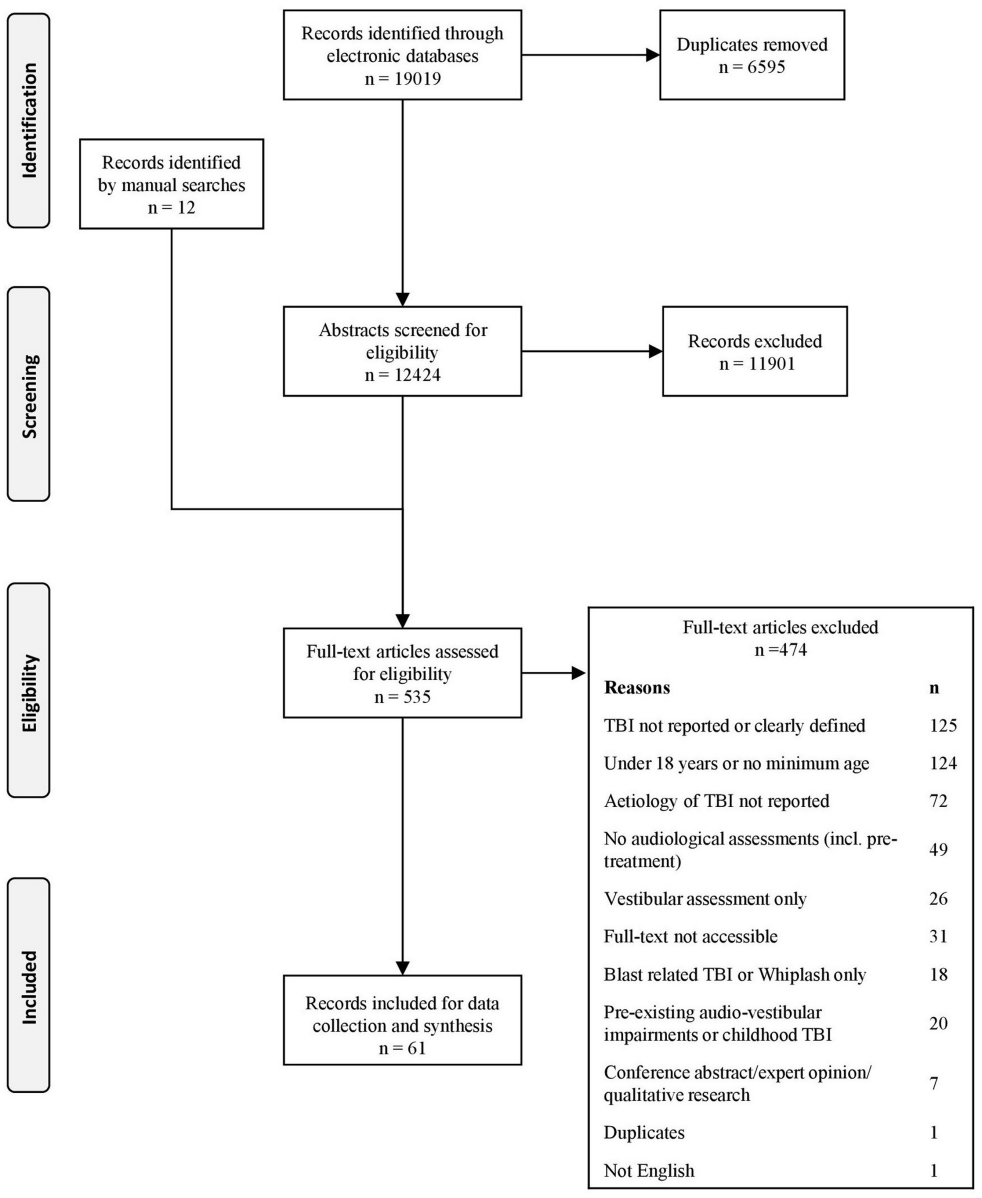
The PRISMA flow diagram for the study selection process.

Figure 1 illustrates the process of record identification and selection. Electronic searches resulted in an initial set of 19.019 records. Duplicates were removed and of the remaining 12.424 records, 11.901 were excluded because the title and abstract indicated that the articles did not meet the eligibility criteria. Manual searches identified a further 12 potential articles which were subjected to full-text screening. Of the remaining 535 records, 474 records were excluded at the full-text screening. Most commonly the studies excluded did not report TBI or clearly define TBI, included participants under 18 years old and did not report TBI aetiology. Full-text records could not be located for 31 records. None of these records could be traced, regardless of support from the University of Nottingham librarian. The electronic and manual searches created a final list of 61 eligible full-text records for data collection.

### 3.1 Study characteristics

Table 1 provides an overview of the study and participant characteristics. As shown by Table 1, the majority of records were reporting case reports/case series (51/61) (11, 22–71) and were mainly conducted in the United States (*n* = 23), the United Kingdom (*n* = 5), Japan (*n* = 4), and Korea (*n* = 3). Articles were published from 1956 to 2023.

**Table 1:** Characteristics of included studies.

Ref	Country	Study Design	Research Aim	Sample Size and age range (years)	Gender	Severity of TBI	Criteria of severity	Fall	MVA	Assault	Sports injury	Multiple	Other	Time of audiological assessment	Pre-TBI status
Eliyas et al. (66)	India	Case report	This report is about a patient with DAI secondary to MVA, where a complete audiological test battery was done	1 (35 yrs)	M	NR	NR		✓					6.5 mths	NR
Ouhbi et al. (67)	Morocco	Case report	To report a case of a young male who developed immediate bilateral facial and left abducens paralysis following a motor vehicle accident, which was managed conservatively with gratifying results	1 (28 yrs)	M	NR	NR		✓					Immed. (f/u: 6 mths)	NR
Lew et al. (46)	USA	Case report	To report the use of brainstem auditory evoked potential as an objective and non-invasive tool to identify hearing dysfunction in the early stage of recovery for patients with severe TBI	1 (55 yrs)	M	Severe	NR	✓						4/8/10 wks	No cognitive, hearing or communication impairment. Alcohol excess
Kagoya et al. (54)	Japan	Case report	To present a very rare case of stapedial footplate fracture in which the superstructure with part of the footplate was dislocated and adhered to the tympanic membrane	1 (25 yrs)	F	NR	NR		✓					11 mths	Unremarkable medical history
Desmond Cremin (24)	UK	Case report	Cases of ossicular chain damage	3: Cases 11, 14, 15 (49, 35, 36 yrs)*	M	NR	NR		✓ (2)	✓				C11: 13 yrs. C14: 18 mths C15: NR	NR
Vong and Daud (62)	Malaysia	Case report	To report a patient with contralateral profound hearing loss and subdural haemorrhage secondary to unilateral petrous part of temporal bone fracture with facial nerve palsy following a high impact head injury.	1 (44 yrs)	M	NR	NR		✓					NR	NR
Pollaers et al. (64)	Australia	Case report	To illustrate a case of severe ossicular chain injury and extrusion of the incus from the middle ear though the tympanic membrane to lie within the external auditory canal	1 (24 yrs)	M	Severe	GCS: 7		✓					3 wks	NR
Habib et al. (60)	Saudi Arabia	Case report	To present bilateral facial paralysis with loss of taste sensation and hearing impairment	1 (23 yrs)	M	NR	NR		✓					13 dys	No history of dysphagia, aspiration or respiratory distress was obtained
Türk et al. (69)	Türkiye	Case report	To present a case of post-traumatic pneumolabyrinth secondary to temporal bone fracture without hearing loss improvement	1 (45 yrs)	F	NR	GCS: 15		✓					Immed.	No clinical history besides polycystic kidney disease and hepatic cysts
Cevette and Bielek (37)	USA	Case report	To present the usefulness of TEOAEs and DPOAEs to further evaluate cochlear function in a patient with traumatic brain injury (TBI) who was subsequently inconsistent in response to sound	1 (NR age-young adult)	F	Severe	NR		✓					1 yr	NR
Hu et al. (40)	Taiwan	Case report	To report a patient with traumatic brainstem contusion, with injury to bilateral, lateral, and caudal inferior colliculus symmetrically	1 (48 yrs)	F	NR	NR		✓					10 dys	NR
Wang et al.(43)	Taiwan	Case report	To present two patients with traumatic conductive deafness who gained significant hearing improvement after incudostapedial joint reconstruction by exploratory tympanotomy to alert clinicians to this treatable entity	2: Cases 1, 2 (27, 22 yrs)	F	NR	GCS: E1M4VE		✓					C1: Immed. C2: 2 mths	NR
Rao et al. (70)	USA	Case report	To present a rare and unique case of headache and hearing loss that illustrates sequalae of traumatic temporal bone fracture, as well as the value of clinical history and heightened clinical concern for an occult, easily overlooked region during imaging	1 (76 yrs)	F	NR	NR	✓						2 days	Sudden light-headedness caused syncope and a fall, resulting in TBI. History includes hypertension and diabetes
Ghorayeb et al. (31)	USA	Case report	To discuss our experience with temporal bone fractures and present three illustrative case reports	1: Case 2 (20 yrs)*	M	Concuss^	NR	✓						24 h	NR
Musiek et al. (47)	USA	Case report	It demonstrates (1) auditory deficits can be a sequel to minor head injury (2) that these deficits are often subtle and may not be detected unless central auditory testing is conducted, and (3) that these deficits may be amenable to remediation	1 (41 yrs)	F	Mild	NR	✓						13 mths	NR
Hugdahl et al. (33)	Norway	Case report	To report a case of auditory hemispatial neglect after a traffic accident resulting in a diffuse lesion in the right frontal lobe and a restricted lesion in the right pulvinar	1 (22 yrs)	M	Severe	NR		✓					4 yrs	NR
Atkin et al. (45)	UK	Case report	To present a case of bilateral sensorineural hearing loss due to bilateral temporal bone fractures following an epileptic seizure	1 (37 yrs)	M	NR	GCS: 11	✓						NR	Secondary generalized epilepsy since age 13; not wearing a protective helmet
Johkura et al. (41)	Japan	Case report	To report a patient with a small midbrain lesion whose auditory dysfunction mimicked auditory agnosia due to bitemporal disorders	1 (46 yrs)	M	NR	NR				✓			6 mths	NR
Shibata (68)	Japan	Case report	To present a case of delayed traumatic intracerebral hematoma (DTICH) presenting as cortical deafness.	1 (60 yrs)	M	NR	NR	✓						1 mth	History of hypertension and atrial fibrillation; on antihypertensive medication and warfarin
Dailey and Barsan (35)	USA	Case report	NR	1: Case 2 (28 yrs)*	M	NR	GCS: 7				✓			Immed. (f/u: 3 dys)	NR
Scott et al. (42)	USA	Case report	To report additional clinical data indicating that audiograms with single and double sensorineural notches in the mid-frequency region may be related to head trauma	1 (37 yrs)*	M	NR	NR		✓					3 mths	Pre-injury audiometry (Figure) showed air conduction thresholds of 0–10 dB HL (0.25–4 kHz) and 0–20 dB HL (8 kHz), within the normal range
Jang et al. (63)	Korea	Case report	To report on a patient with sensorineural hearing loss who showed injury of auditory radiation following mild TBI, demonstrated by diffusion tensor tractography	1 (35 yrs)	F	Mild	GCS: 15		✓					1.5 yrs	NR
McKennan and Chole (32)	USA	Case report	To report the unusual features and method of management of post-traumatic cholesteatoma	2: Cases 1,3 (23, 28 yrs)*	M = 1 F = 1	C1: Concuss^ C3: Concuss^	NR	✓	✓					C1: Immed. (f/u:2 wks) C3: 7 yrs	NR
Brookes and Graham (30)	USA	Case reports	NR	3: Cases 1–3 (76, 20, 37 yrs)	M = 2 F = 1	NR C2: Severe	NR		✓ (2)	✓				C1: 6 mths C2: 3 yrs. C3: 2 mths (f/u: 4 yrs)	NR
Kreuzer et al. (57)	Germany	Case report	To report the case who developed severe chronic tinnitus after a pronounced TBI leading to depressive symptoms and alcohol addiction and who was treated successfully with repetitive transcranial magnetic stimulation.	1 (53 yrs)	M	Severe	NR	✓						4 yrs	NR
Majmundar et al. (49)	USA	Case report	To discuss the clinical aspects and management of traumatic cholesteatomas of the temporal bone	1 (21 yrs)	M	Moderate or Severe	NR		✓					NR	NR
Jeon et al. (58)	Korea	Case report	To report satisfactory experience of multichannel cochlear implantation in the bilateral transverse temporal bone fractures with severe brain damage	1 (33 yrs)	M	Severe	NR		✓					12 yrs	NR
Nagapoornima et al. (71)	India	Case report	To report a follow up of a patient with TBI; hearing loss, speech understanding difficulty and tinnitus being the main complaints	1 (23 yrs)	M	Severe	GCS: 8		✓					Immed. (f/u: 1.5/2/2.5 yrs)	No history of seizures, vomiting, headache, CNS bleed or ear bleed
Feneley and Murthy (36)	UK	Case report	To describe the case who presented with acute bilateral deafness and vestibular dysfunction following occipital bone fracture	1 (57 yrs)	M	NR	NR	✓						3 dys (f/u: 3 wks)	Previously in excellent health, with no medications and no history of excessive alcohol consumption
Bertholon et al. (48)	France	Case report	To report cases who complained of positional vertigo shortly after head trauma	1: Case 1 (19 yrs)*	M	NR	NR	✓						1 mth	Case 1: No significant medical history
Ylikoski et al. (28)	USA	Case report	To search for pathologic changes indicating nerve injury by examining the operative specimens of the eighth nerve from patients with post-traumatic dizziness and combining these findings with the clinical, otologic and surgical features of each case, to determine the site of primary lesion	2: Cases 8–9 (55, 53 yrs)*	M	C8: NR C9: NR	NR		✓ (2)					NR	NR
Roup et al. (11)	USA	Case report	To present a case report of a patient with a history of TBI, including self-perceived hearing difficulties and poorer-than-normal auditory processing performance	1 (58 yrs)	F	Mild	NR		✓					12 mths	No hearing or listening problems
Fitzgerald (38)	USA	Case report	To discuss the typical history and diagnostic tests for patients with perilymphatic fistula	1: Case 1 (28 yrs)*	F	NR	NR		✓					6 dys (f/u: 10 wks)	NR
Fujimoto et al. (50)	Japan	Case report	To report a rare and informative case of bilateral progressive sensorineural hearing loss after traumatic subarachnoid haemorrhage and brain contusion, in which cochlear implantation was very successful.	1 (55 yrs)	M	NR	NR	✓						1 mth (f/u: 11/13/15/23 mths)	No history of administration of ototoxic agents, including aminoglycosides
Ottaviano et al. (53)	Italy	Case report	To report two cases of sensorineural hearing loss with benign paroxysmal positional vertigo and anosmia following traumatic head injury	1: Case 2 (57 yrs)*	F	NR	NR		✓					7 mths	NR
Kanavati et al. (61)	UK	Case report	NR	1 (24 yrs)	M	NR (GCS:12)	NR			✓				NR	NR
Jani et al. (34)	USA	Case report	To report the usefulness of magnetic resonance imaging and auditory brainstem evoked responses in diagnosis	1 (46 yrs)	F	Moderate or Severe	NR		✓					13 dys (f/u: 17 dys)	History of major mood disorder
Schuknecht and Davison (22)	Canada	Case report	NR	4: Cases 1–3, 5 (23, 29, 29, 21 yrs)*	M	NR	NR	✓	✓ (3)					C1: 2 dys (f/u: 2/4wks) C2: 24 h (f/u: 4 mths) C3: 2 yrs. C5: 48 h	NR
Waninger et al. (59)	USA	Case report	To describe a unique mechanism of ear barotrauma (intratympanic haemorrhage) and concussion caused by helmet-to-helmet contact in American football	1 (26 yrs)	M	Concuss^	NR				✓			36 h	No history of previous concussions or head/ear injuries
Preber and Silversklöld (23)	Sweden	Case report	NR	4: Cases 1–3, 5 (36, 48, 57, 53 yrs)*	M = 2 F = 2	NR	NR	✓	✓ (3)					C1: 1 mth C2: 3 yrs. C3: 3 mths C5: 1 yr	NR
Sousa Menezes et al. (65)	Portugal	Case report	To report the case of a patient with pneumolabyrinth, involving both the vestibule and the cochlea with intense vestibular symptoms, in whom the anatomic defect was evident on surgical exploration and successfully managed surgically	1 (52 yrs)	M	NR	NR	✓						3 dys	No relevant personal history
Lerut et al. (51)	Belgium	Case report	To discuss the case and the final diagnosis of carotico-cavernous fistula	1 (68 yrs)	F	NR	NR	✓						2 dys (f/u: 2 mths)	NR
Durbec et al. (56)	France	Case report	NR	1 (22 yrs)	M	NR	NR			✓				8 dys	NR
Lyos et al. (39)	USA	Case report	To describe three patients with transverse temporal bone fracture who presented with residual auditory function only to develop profound sensorineural hearing loss	3: Cases 1–3 (20, 20, 26 yrs)	M	NR	NR	✓		✓			✓	C1: Immed. (f/u: 1 wk) C2: 3 mths C3: 5 days	NR
Tonkin and Fagan (26)	Australia	Case report	The case histories of thirteen patients with such a fistula are described	4: Cases 7–10 (20, 44, 55, 26 yrs)*	M	NR	NR	✓ (3)		✓				C7: several wks C8: 7 mths C9: 5 mths C10: NR	C9: Diabetic underwent a right below the-knee amputation
Paparella and Mancini (29)	USA	Case report	To describe representative case reports, from the clinic and from temporal bone pathology laboratory, of the patients with post-traumatic Meniere’s syndrome in the absence of temporal bone fracture	2: Cases 1, 11 (21, 60 yrs)*	M = 1 F = 1	NR	NR	✓	✓					C1: NR C11: 3 yrs	NR
Mohd Khairi et al. (52)	Malaysia	Case report	To illustrate patients who sustained extradural haemorrhage following a motor vehicle accident with profound sensorineural deafness on the opposite ear	1: Case 1 (31 yrs)*	M	NR	NR						✓	NR	NR
Gluncić et al. (44)	Croatia	Case report	To describe the management and recovery of the patient with a stab wound of the temporal region caused by a knife. The treatment of the wound required multidisciplinary approach.	1 (56 yrs)	M	NR (GCS:14)	NR			✓				7 dys	NR
Jacobs et al. (27)	USA	Case report	To present results of surgical repair in three patients with fistulas	1: Case 1 (59 yrs)*	F	C1: NR	NR						✓	2 mths	NR
Chung et al. (55)	Korea	Case report	To present the case with bilateral otic capsule violating temporal bone fractures due to head trauma	1 (44 yrs)	M	NR	NR	✓						6 wks	NR
Frew (25)	UK	Clinical records	NR	1 (18 yrs)	F	NR	NR		✓					Immed. (f/u: 1/2 yrs)	NR
Knoll et al. (82)	USA	Prospective study	(1) investigate the presence of auditory symptoms in patients with TBI with normal hearing and (2) their impact on audiometric quality-of-life indicators	31 (19–64 yrs)	M = 1 F = 21	Mild (n22) Moderate–Severe (n9)	Mayo TBI severity classification (112)	✓	✓	✓	✓	✓		Mean: 70.1 (±53.1) mths	No significant audiovestibular signs, symptoms, pathologies, no significant noise exposure and no neurological and psychiatric history
Knoll et al. (74)	USA	Prospective study	To determine the auditory symptomology and the impact of these symptoms on quality-of-life in patients with a history of non-blast mTBI.	52 mTBI (19–81 yrs) 55 Control^1^ (18–80 yrs)	mTBI: M = 14, *F* = 38 Control: M = 24, *F* = 31	Mild (n52)	ACRM criteria (113)	✓	✓	✓	✓	✓	✓	Range: ≤ 1–96 mths	No significant audiovestibular signs, symptoms, pathologies, no significant noise exposure and no neurological and psychiatric history
Motin et al. (80)	Israel	Prospective study	To identify patients with BPPV among patients with severe TBI and to evaluate the effectiveness of the Particle Repositioning Maneouvre	20 (19–61 yrs)	M = 1 F = 2	Severe (n20)	NR	✓	✓					Mean: 67 (±14) dys	No history of vertigo or pre-existing inner ear disease
Bunt et al. (94)	USA	Prospective study	To examine differences in concussion symptom reporting between female and male adults considering current psychological symptoms such as anxiety and depression and pre-injury factors in order to identify sex differences which may guide treatment efforts.	132 (19–78 yrs)	M = 5 F = 80	Concuss^	GCS: 13–15	✓	✓	✓	✓		✓	Mean: 13.9 dys-F Mean: 11.9 dys-M	Previous concussion (n45)
Jafarzadeh et al. (78)	Iran	Prospective cross-sectional	The vestibular assessment of patients with persistent symptoms of mTBI by different vestibular tests	21 (18–60 yrs)	M = 2 F = 1	Mild	GCS: 13–15	✓						118.2 ± 52.5 dys	No history of hearing loss, vertigo, imbalance or gait abnormality
Attias et al. (72)	Israel	A cross-sectional design	To explore the function of the auditory system in TBI patients with and without Acs but having normal pure-tone audiograms	24 TBI w/ACs (20–52 yrs) 10 TBI w/o ACs (22–43 yrs) 15 Control^2^ (22–42 yrs)	TBI+: M = 22, F = 2 TBI-: M = 8, F = 2 Control: M = 7, *F* = 8	Mild (n8) Moderate–severe (n26)	Compound score^3^	✓	✓		✓		✓	NR	NR
Gard et al. (75)	Sweden	Observational study	To establish the cause of vestibular impairment in athletes with concussion who have PPCS	21 sports- concussion 21 control^4^ (18–43 yrs)	SRC: M = 14, F = 7 Control : M = 11, F = 10	Concuss^	NR				✓			Mean: 2.5 yrs	History of at least one sports-related concussion. No previous or current self-reported neurological or psychiatric disorders
Hoover et al. (73)	USA	A matched group design	Deficits, understanding speech in a background of speech noise following mTBI were evaluated with goal of comparing the relative contributions of peripheral auditory, auditory processing, and nonauditory cognitive factors	11* mTBI (25–71 yrs) 9 Control^5^ (18–24 yrs) 11 Match^6^ (27–70 yrs)	NR	Mild	DSM-5^7^	✓	✓		✓	✓		Range: 1–46 yrs	NR for mTBI group
Ishai et al. (81)	USA	Otopathology study	To evaluate the cochleae of patients who sustained head trauma w/o temporal bone fracture to better understand associated histopathology that may give rise to auditory dysfunction.	3: Cases 2, 3, 5 (71, 66, 72 yrs)*	M = 2 F = 1	C2: Concuss^ C3: NR C5: NR	NR	✓ (2)	✓					Range: 2–12 yrs	No significant audio-vestibular signs, symptoms, pathologies, no significant noise exposure C2: Myoclonic seizures since childhood
Hegel and Martin (76)	Lebanon	Behavioral treatment study	To describe the evaluation and behavioral treatment of a gentleman with pulsatile tinnitus	1 (37 yrs)	M	NR	NR		✓					4 yrs	NR

* Sample size in original was larger.

^1 Consisted of individuals from a sports medicine clinic. No prior history of TBI and otologic disorders.^

^2 No TBI and normal hearing.^

^3 Mild TBI was defined as loss of consciousness < 10 min. or amnesia, GCS of 13–15, no skull fracture on physical examination. Severe TBI was defines as coma lasting more than 6 h, GCS 8 or less and with neurological deficits.^

^4 Healthy athletes with no previous SRC and exercising three times per week.^

^5 No history of TBI or other neurological disorders and normal pure-tone thresholds.^

^6 Age and pure-tone thresholds matched the listeners in the mTBI group.^

^7 Patient report of an insult to the head resulting in a period of confusion or disorientation, posttraumatic amnesia of any duration, and loss of consciousness less than 30 min.^

ACs, auditory complaints; ACRM, american congress of rehabilitation medicine; BPPV, benign paroxysmal positional vertigo; C, case; Concuss^, concussion; DAI, diffused axonal injury; DSM-5, diagnostic and statistical manual of mental disorders; F, female; f/u, follow-up; GCS, glasgow coma scale; Immed., immediate; M, male; MVA, motor vehicle accidents; mTBI, mild TBI; NR, not reported; PPCS, persisting post-concussive symptoms; SRC, sports-related concussion.

### 3.2 Participant characteristics

Across 61 records, 507 participants were included. Of these, 396 were in the patient group, whilst for four studies, 111 participants were in the control groups (either without TBI or without both TBI and auditory symptoms) (72–75). Pre-TBI health status of participants was not reported consistently across studies (Table 1). Assessment time since injury varied widely across studies (Table 1). In 39 studies, follow-up/s’ assessments were performed after the initial time of injury before any treatment was offered (22–27, 30, 32–40, 42–44, 46–51, 53–55, 57, 58, 63–65, 67–71, 76).

### 3.3 Overview of auditory impairments following non-blast related TBI

Many different symptoms such as hearing loss, tinnitus, and hyperacusis were reported across the studies. These symptoms were assessed using a variety of tests, including peripheral and central auditory function assessments and patient-reported outcome measurements (PROMs) which are briefly described below. A summary of these tests and PROMs are presented in Supplementary Appendix Table 3 and the results are shown in Table 2.

**Table 2:** Audiological findings of included studies.

Ref	Gender	Severity of TBI	Fall	MVA	Assault	Sports injury	Multiple	Other	Patients reported auditory symptoms	Hearing	Difficulties understanding speech	Tinnitus	Hyperacusis
Schuknecht and Davison (22)	M (4)	NR	✓ (1) C5	✓ (3) C1 C2 C3					C1: B/L HL C2: RE HL C3: RE Profound deafness C5: LE HL	C1: 2 dys after: **Otological exam:** Dried blood in both EAC, blood filled middle ears **Rinne:** B/L negative 2 wks later: **PTA:** B/L C-SN HL 4 wks after TBI: Normal TM CHL disappeared but the SNHL remained C2: Next, dy: **Otological exam.:** RE serohemorrhagic fluid, moderately severe combined C-SN HL 4 mths after TBI: **PTA:** RE Mild CHL worse for HFs, LE Normal C3: **PTA:** RE profound deafness, LE slight SNHL at LFs, severe loss for HFs C5: 48 h after: **Otological exam.**: Normal TMs **PTA:** LE SNHL **Weber:** Lateralization to RE for all frequencies **ABLB (Loudness recruitment):** present around 500 Hz			
Preber and Silversklöld (23)	M = 2 F = 2 C1: M C2: F C3: M C5: F	NR	✓ (1) C5	✓ (3) C1 C2 C3					C1: NR auditory symptoms C2: NR auditory symptoms C3: NR auditory symptoms C5: NR auditory symptoms	C1: 1 mth after: **Neuro-otologic exam.:** Normal **PTA:** B/L Slight SNHL C2: 3 yrs. after: **Neuro-otologic exam.:** Normal **PTA:** Normal C3: Haemorrhage from the LE 3 mths after: **PTA:** B/L SNHL C5: 1 yr. after: **Neuro-otologic exam.:** Normal **PTA:** Normal			
Frew (25)	F	NR		✓					Deafness in the LE 2 yrs. after (July 1966) A return LE deafness	Normal TMs **PTA:** LE SNHL 1 yr. after (October 1965): Returned to normal hearing 2 yrs. after (July 1966): **PTA:** LE CHL			
Scott et al. (42)	M	NR		✓					NR	3 mths after: **PTA:** RE 2000 Hz SN notch. LE mild HF SNHL at 4000 to 8,000 Hz			
Ottaviano et al. (53)	F	NR		✓					Case 2: B/L HL	**Otoscopy:** Normal 1 mth earlier in another institution: **PTA:** B/L Moderate SNHL Further assessment: **PTA**: B/L SNHL **SA (SRT**_**1**_**):** 20 dB in RE and LE **ABR:** showed the cochlear origin of B/L HL			
Jeon et al. (58)	M	Severe		✓					LE deafness 4 yrs. later, sudden deafness in RE, no recovery over 2 yrs. of follow-up.	6 yrs. after: **Physical exam.**: Both intact external auditory canals & TMs **PTA:** B/L profound SNHL **ABR:** B/L absent			
Vong and Daud (62)	M	NR		✓					NR	**ENT exam.:** RE hemotympanum with intact TM **PTA:** LE profound SNHL, RE mild to severe CHL			
Ishai et al. (81)	C2: F C3: M C5: M	C2: Concuss^ C3: NR C5: NR	✓ (2) C2 C3	✓ (1) C5					NR	C2: LE profound SNHL C3: RE Mild to severe down-sloping, mild SNHL C5: LE Mild to severe down-sloping, moderate SNHL			
Jang et al. (63)	F	Mild		✓					HL ~ 2 wks post-head trauma, worsening over time. ~ 1.5 yrs. post-trauma, severe HL	**Physical exam.:** B/L no abnormality **SA:** NR in detail, similar to PTA **PTA:** Moderate SNHL & Severe SNHL			
Pollaers et al. (64)	M	Severe TBI		✓					RE HL	Clotted blood & debris obscruing in TM **Weber**: lateralised to the RE, BC better AC in RE, AC better BC in LE **PTA:** LE mild low-frequency SNHL RE moderate to severe CHL **Tymp:** LE Type C, RE unobtainable			
Eliyas et al. (66)	M	NR		✓					Diff understanding speech & slurred speech	**PTA:** RE Mild SNHL, LE Moderate SNHL **Tymp:** B/L As type **TF:** Rinne positive B/L, Weber lateralising RE **SDS:** 0% **TD:** Retrocochlear pathological findings **DPOAE:** Absent in both ears **ABR:** No V peaks. I & III peaks replicable in both ears **LLR:** Normal absolute latencies in both ears **MMN:** No peak **P300:** No peak			
Ouhbi et al. (67)	M	NR		✓					Deafness	**Otoscopy:** B/L hemotympanum **PTA:** RE total HL, LE MHL **Tymp:** B/L flat curves **AR:** Abolished on both sides 6 mths: Partially improved hearing **PTA:** RE Moderate SNHL, LE Mild SNHL			
Desmond Cremin (24)	M	NR		✓ (2) C11C15	✓ (1) C14				C11: RE deafness C14: Tinnitus C15: Deafness	*PTA results not reported for C11, only presented as figures PTA*: C11: A decrease from 1 kHz to 8 kHz & from 25 dB HL to 60 dB HL in RE. Air- bone gap 10 dB at 1kHZ & 35 dB at 4 kHz, CHL C14: MHL C15: MHL			
Brookes and Graham (30)	C1: M C2: F C3: M	NR C2: Severe		✓ (2) C2 C3	✓ (1) C1				C1: RE deafness C2: Severe LE deafness. After 3 yrs., left otolgia C3: A mild right deafness & ti 4 yrs. later: Aural blockage	C1: RE fresh blood **PTA:** RE CHL C2: **Exam.:** LE purulent drainage from the deep ear canal **PTA:** LE total deafness C3: Over 2 mths: **Otologic exam.:** A step-off fracture in the deep part of the superior ear canal. **PTA:** Mixed deafness 4 yrs.: **Otoscopy:** An active cholesteatoma **PTA:** Mild CHL with a high-tone SN component			
McKennan and Chole (32)	C1: F C3: M	C1: Concuss^ C3: Concuss^	✓ C3	✓ C1					C1: NR C3: NR	C1: LE cerebrospinal fluid otorrhea **PTA:** B/L CHL Over 2 wks: RE CHL **SA: (SRT**_**2**_**):** LE 55, (**SDS):** 76% at 90 dB C3: RE purulent & bloody otorrhea **PTA:** Mild CHL **SA: (SRT**_**2**_**):** RE 20, **(SDS):** 100% at 55 dB			
Wang et al. (43)	F (2)	NR		✓ (2)					C1: RE HL C2: LE HL	C1: **PTA:** RE CHL Intact eardrum, no sign hemotympanum **AR:** LE normal, RE absent **Tymp:** Type Ad C2: Intact eardrum, no sign hemotympanum **PTA:** LE CHL **Tymp:** Type Ad			
Majmundar et al. (49)	M	Mod-Severe		✓					NR complaints immed. Post-TBI 2 yrs. later, the patient presented to the emergency department with a 5-dys history, right otalgia, HL on the RE	Mild CHL			
Kagoya et al. (54)	F	NR		✓					LE HL	Cerebrospinal fluid otorrhea 11 mths after: **PTA:** LE CHL **Tymp:** Ad type **AR:** Left positive with right-sided stimuli			
Habib et al. (60)	M	NR		✓					Hy B/L HL	RE bloody otorrhea **PTA:** Both ears MHL (LE better than RE) Hearing improvement over time			
Paparella and Mancini (29)	M = 1 F = 1 C1: F C11: M	NR	✓ (1) C11	✓ (1) C1					Case 1: NR Case 11: LE HL & Ti	*RE & LE air-conduction results were presented on the audiogram, but no explanation was given regarding the results. C1: **PTA:** RE Normal, LE HL C11: **PTA:** B/L HL increasing toward HFs			
Hugdahl et al. (33)	M	Severe TBI		✓					NR	**Audiometer screening:** Normal within the critical frequency range **DL-NF:** RE recall 86%, LE recall 5% **DL-FR:** RE recall 93%, LE recall 3% **DL-FL:** RE recall 86%, LE recall 2% **MT:** RE recall 100%, LE recall 83% Diagnosis of auditory attentional neglect			
Fitzgerald (38)	F	NR		✓					C1: NR	C1: 6 dys after: Dried blood in RE EC 10 wks after: **PTA:** Normal in all frequencies **ECOG:** RE abnormal			
Roup et al. (11)	F	Mild TBI		✓					Hy, Ti, & trouble hearing in background noise	**Otoscopy:** B/L normal **Tymp:** B/L normal **AR**: B/L present **PTA:** B/L normal (≤ 25 dB HL) **SA (SRT**_**2**_**):** NR; **WRS-Q (SDS):** RE excellent (92%), LE (100%) **HHI-A:** Substantial severity (score 96 out of 100) **SCAN-3A**: Age-appropriate skills for auditory closure, auditory figure-ground, binaural separation, temporal processing) Abnormally low performance for competing words, binaural integration **500 Hz MLD:** Normal **GIN:** RE normal, LE abnormally poor	**QuickSIN:** Mild SNR loss of 6.5 dB **1–2 pair DDR:** Normal **3 pair DDR:** RE Normal, LE below normal **R-SPIN:** Excellent at high-predictability sentences Abnormally poor at low-predictability sentences		**HQ:** all sounds too loud, ranging from the vacuum cleaner to music in grocery stores
Ylikoski et al. (28)	M (2)	NR		✓ (2)					C8: Severe the RE HL C9: RE HL & Ti	C8: **PTA:** Severe HL (a falling curve 93 dB level) **Fowler (ABLB):** No recruitment at low frequencies C9: **PTA:** RE HL (level of 90 dB)			
Hegel and Martin (76)	M	NR		✓					Ti worsened when lying down Pulsatile Ti accompanied HL Pulsing sound audible on auscultation of the area	LE totally deaf, RE moderate to severe HL		**LS:** NR rating score	
Nagapoornima et al. (71)	M	Severe		✓					B/L HL & Ti	Dec 2019: **PTA:** Not follow instructions well **SA:** could not be tested **Otoscopy:** B/L normal & intact TMs June 2020: **PTA:** RE Moderate HL, LE Mild HL **SA:** could not be tested Oct 2020: **PTA:** B/L mild HL **SA (SRT**_**1**_**):** RE 50, LE 40, (SDS): RE 28%, LE 52% **Tymp:** B/L Type A **AR:** B/L present, except 4 kHz contra **TEOAE:** B/L present **DPOAE:** B/L present upto 3 kHz **ABR:** B/L III&V upto 80 dB nHL **LLR:** Within normal limits **HHI-A:** Severe handicap (score:40) **SCAP-A:** At risk for APD Mar 2021: **PTA:** B/L mild HL **SA (SRT):** RE 45, LE 45, **(SDS):** RE 45, 55% June 2021: **PTA:** RE Mild HL, LE Minimal HL **SA (SRT**_**1**_**):** RE 45, LE 55, **(SDS):** RE 45%, LE 80% **Tymp:** B/L Type A **AR:** B/L present, except 4 kHz contra **TEOAE:** B/L present **DPOAE:** B/L present RE upto 4 kHz, LE upto 3 kHz **ABR**: RE III upto 50 dB nHL, LE III upto 40 dB nHL RE V upto 80 dB nHL, LE V upto 45 dB nHL		Oct 2020: **THI score**: 94 (Catastrophic)	
Türk et al. (69)	F	NR		✓					Left aural fullness and total HL on the LE	LE hemotympanium **PTA:** LE total HL Permanent HL			
Jani et al. (34)	F	Mod- severe		✓					Unable to hear for several days	13^th^ dys: **TF:** No hearing, feeling vibration 17^th^ dys: **PTA:** B/L static HL between 50–90 dB for pure-tone & speech stimuli. **Tymp:** B/L normal **AR:** B/L present uncrossed, absent crossed **ABR:** LE present waves I to IV, RE present waves I to III, B/L absent wave V B/L severe peripheral HL and brainstem dysfunction			
Cevette and Bielek (37)	F	Severe		✓					Severe communicative deficits	**PTA** *(at another centre):* RE moderate to severe HL, LE severe to profound HL **Informal speech testing (SRT**_**1**_**):** No response; Three-choice spondees: one correct response at 75 dB HL **PTA:** No response **Medical Exam.:** No evidence of TB fracture & TM abnormalities **Tymp:** B/L Type A **Ipsi-contra AC**: B/L absent **ABR:** B/L abnormal at 90 dB nHL **TEOAE**: B/L normal to near-normal **DPOAE:** B/L present from 0.1 to 6 kHz except 2 kHz			
Hu et al. (40)	F	NR		✓					Total deafness but able to speak	**TF:** No hearing, feeling vibration **PTA:** No response speech or PT **Tymp:** Normal middle ear pressure & mobility B/L **AR:** Preserved B/L 14^th^ dy of hospital: **ABR:** Normal waveform Wave V well-preserved at increased frequencies & decreased intensities Non-symptomatic deafness			
Tonkin and Fagan (26)	M (4)	NR	✓ (3) C7 C9 C10		✓ (1) C8				C7: Right-sided Ti, and severe HL C8: About two mths postoperatively: a sense of pressure in the LE, LE deafness, constant, LE and Ti C9: Left ringing Ti, severe LE HL C10: RE HL, a fullness in the RE	C7: **PTA:** RE normal up to 3 kHz & severe SNHL above 3 kHz C8: 7 mths after: **PTA:** LE SNHL C9: 5 mths after: **PTA**: SNHL **SA (SDS):** Not performed C10: **PTA:** RE Moderate MHL			
Atkin et al. (45)	M	NR	✓						B/L deafness	**PTA:** B/L SNHL			
Lew et al. (46)	M	Severe TBI	✓						NR	4 wks after: **PTA:** Inconclusive **TF:** Inconclusive 8 wks after: **ABR:** Not elicited B/L up to 85 dB nHL 10 wks after: **Otoscopy:** Normal **Tymp:** Normal **PTA:** B/L Profound SNHL			
Fujimoto et al. (50)	M	NR	✓						RE HL LE HL 4 days post-trauma, with B/L impairment worsening 11 mths post-trauma: worsening in RE	1 mth after: B/L normal eardrums **Tymp**: B/L Type A **PTA:** B/L SNHL; RE 68 dBHL, LE 73 dBHL **AR (contra):** RE present (at 100–110 dB at 500 Hz, 1,000 Hz, 2000 Hz), LE absent **Bekesy’s test:** Jerger Type I, B/L normal **ABR:** Both sides only present wave V at 80 dB nHL **DPOAE:** Both sides very poor; severe inner ear damage **SA (SDS):** RE 10%, LE 15% at 90 dB HL 11 mths after TBI: **PTA:** RE 88 dB HL, LE 80 dB HL 13 mths after TBI: **PTA:** RE more than 105 dB HL, LE 88 dB HL 15 mths after TBI: **PTA:** RE 97 dB HL, LE 92 dB HL 23 mths after TBI: **PTA:** RE 100 dB HL, LE 92 dB HL (Severe to profound HL) **SA (SDS):** RE 5%, LE 10% at 100 dB HL **ABR**: Both sides absent			
Sousa Menezes et al. (65)	M	NR	✓						Sudden LE HL, otalgia, otorhea	**Otoscopy:** B/L normal **TF:** Weber lateralize to RE, Negative Rinne LE **PTA:** RE normal, scotoma at 4 kHz, LE Profound SNHL			
Bertholon et al. (48)	M	NR	✓						C1: No definite hearing complaint	C1: Almost 1 mth after: **PTA:** A slight RE HF SNHL			
Feneley and Murthy (36)	M	NR	✓						Total deafness	**Otoneurological exam.:** Normal EAC, TM 3 dys after: **PTA:** B/L no response **ABR**: B/L no response at max. Stimulation **Tymp:** Normal 3 wks after TBI: **PTA:** Recovery to 60 dB at 250 Hz, sharply decline, NR beyond 1 kHz, B/L Severe SNHL			
Jafarzadeh et al. (78)	M = 20 F = 1	Mild	✓						HL (n9) Ti (n4)	**Otoscopy:** Normal **PTA:** HL trauma caused in 10/21 (47.6%) Mild HF to B/L Profound SNHL Symmetrical SNHL in most cases U/L or asymmetrical SNHL in 4 pts. **SA:** NR **Tymp:** Normal (Type A) for all		Ti in 4 pts. (one had profound SNHL, others had mild and moderate HL) B/L Ti in 2 Pts	
Musiek et al. (47)	F	Mild TBI	✓						Diff w/ comprehension of complex auditory directives, understanding rapid speech & hearing background noise (LE worse than RE) Ti noted post-accident; resolved after a few months in the patients reported auditory symptoms	13 mths after: **PTA:** B/L normal **SA (SRT**_**2**_**):** B/L excellent **DPOAE:** Normal cochlear function **DPT:** Abnormal performance **FPT:** Normal range **MLR:** LE Na-Pa waves larger than RE across electrodes. **ABR:** B/L normal results	**DDT:** Outside of normal range for both ears **TCS:** Outside of normal range for both ears **CST:** Outside of normal range for both ears The greater deficit in LE for all three test		
Chung et al. (55)	M	NR	✓						B/L HL	**Otoscopy:** B/L normal **PTA:** No response at the maximum stimuli **Tymp:** B/L Type A **ABR:** B/L No waves V			
Kreuzer et al. (57)	M	Severe	✓						Ti began ~6 wks post-TBI, described as high-pitched ringing/whistling, B/L and central, with partly pulsatile character	**PTA:** normacusis between 125–2000 Hz with steep decline toward higher frequencies peaking at 55 dB at 8 kHz on both sides. **SA:** LE 85%, RE 95% at 65 dB **Tymp:** B/L normal **AR:** B/L normal		**TQ score:** 67 (extreme Ti severity: grade 4) **NRS score:** 10 4 yrs. later: Ti worsened	
Lerut et al. (51)	F	NR	✓						Left-sided, pulsatile Ti 4 wks after TBI: Left-sided pulsatile Ti	**Clinical exam.:** LE laceration, haemotympanum **PTA:** RE near normal, LE MHL 2 mths after: **PTA**: Persisting air-bone gap		Diagnosis of pulsatile Ti reported to ocular symptoms	
Lyos et al. (39)	M (3)	NR	✓ (1) C2		✓ (1) C3			✓ (1) C1	C1: NR C2: Auditory perception in the RE but not LE After 3 mths, RE HL, LE fluctuating HL C3: RE Severe HL & Ti	C1: **Physical exam.:** LE hemotympanum **TF: (Rinne):** LE BC is better than AC **PTA:** LE MHL 1 wk. after: **Weber**: Lateralizing to RE LE hemotympanum **PTA & SA:** RE Normal, LE No response **AR:** LE Absent C2: 3 mths after: **Otological Asses.:** Slightly retracted TM with B/L serous effusion **PTA:** B/L fluctuating severe to profound MHL C3: 5 dys after: **Physical exam.:** RE a hemotympanum behind an intact TM **PTA:** RE severe SNHL, LE normal			
Ghorayeb et al. (31)	M	Concuss^	✓						C2: HL	C2: U/L ossicular chain disruption			
Shibata (68)	M	NR	✓						B/L HL	1 mth after: **ABR:** Normal response Diagnosed cortical deafness caused by delayed traumatic intracerebral hematoma			
Rao et al. (70)	F	NR	✓						HL	2nd dy of hospital: **Otoscopy:** clean external canal B/L **TF:** RE reduced BC			
Kanavati et al. (61)	M	NR			✓				Complete deafness, Ti	**ENT exam.:** RE hemotympanum **Audiological assessment:** B/L profound SNHL			
Durbec et al. (56)	M	NR			✓				RE HL	**Otoscopy**: Normal **PTA and SA**: RE total deafness, LE normal			
Gluncić et al. (44)	M	NR			✓				NR	**Otoscopy:** No signs 7 dys after hospitalization: **PTA**: LE CHL increasing toward HFs			
Johkura et al. (41)	M	NR				✓			Upon regaining consciousness 5 days post-accident, inability to recognize sounds	**PTA:** Normal thresholds for all frequencies **KDT:** RE: 25%; LE: 20% **ESI:** Only two identified within 16 environmental sounds **TTD:** Correctly in 33% **RPD:** Correctly determined two presented rhythms **MRT:** Unable to say names of familiar songs **SL:** Not localize **ABR:** B/L obtained with low-amplitude, prolonged latency of wave V **MLAEPs/MLR:** Wave Pa was recorded only contralaterally to stimulation			
Waninger et al. (59)	M	Concuss^				✓			Ti	36 h after: **ENT exam.:** RE Intratympanic hemorrhage **PTA:** Normal Diagnosis was intratympanic hemorrhage secondary to barotrauma caused by helmet-to-helmet contact			
Gard et al. (75)	SRC: M = 14, F = 7 Control: M = 11, F = 10	Concuss^				✓			NR	**PTA:** Normal for all pts., except one athlete with SRC had RE HL			
Dailey and Barsan (35)	M	NR				✓			NR	Upon his arrival: **Physical exam.:** RE Blood flowing 3rd dy: **PTA**: Moderate RE CHL (persist)			
Mohd Khairi et al. (52)	M	NR						✓	NR	**ENT exam.:** RE haemotympanum **PTA:** RE SNHL			
Jacobs et al. (27)	F	NR						✓	C1: NR auditory symptoms	C1: **Neuro-otological exam.:** RE moderate SNHL 2 mths after the injury: **PTA**: Improvement SNHL			
Attias et al. (72)	TBI w/ ACs.: M = 22, F = 2 TBI w/o ACs: M = 8, F = 2 Control: M = 7, F = 8	Mild TBI (n8) Moderate–severe TBI (n26)	✓ (2)	✓ (30)		✓ (1)		✓ (1)	ACs incl Ti, diff hearing in noise, and Hy (n24)	**Otoscopy:** NR **Tymp:** NR **PTA:** Mean (all grps): Normal **TEOAE:** Lower amplitude in all TBI grps vs. controls; Higher amplitude in TBI w/ACs grp vs. w/o ACs **MOSE:** Absent in one or both ears in 87% of the TBI Pts w/ ACs			TBI w/ ACs grp: 5 ears not tested with TEOAE due to hypersensitivity to acoustic stimulation
Motin et al. (80)	M = 18 F = 2	Severe	✓	✓					NR	**PTA:** RE SNHL in one pts. Others had normal hearing **ABR:** Normal			
Hoover et al. (73)	NR	Mild	✓ (3)	✓ (3)		✓ (3)	✓ (2)		NR	**Otoscopy:** NR **Tymp:** Normal **PTA:** Mean (all): normal hearing (15.8). mTBI grp: small range of HL **SRT**_**1**_: Mean (14.2) = normal **WRS-Q (SDS):** mean = 95% **TFS:** 7 mTBI pts.: abnormal **SRR:** mTBI grp: outside of the normal range **IPD:** 5 mTBI pts.: outside of the normal range **IC:** 4 mTBI pts.: Impaired **SSQHS**: NR	mTBI grp: No diff in quiet, all report diff in noise **QuickSIN:** 6/11 mTBI pts.: abnormal at least one ear **WIN**: 8/11 mTBI pts. abnormal at least one ear **SRM:** Reduced in mTBI grp		
										**Effect of aetiology:** Fall: Small range HL 2/11 pts. MVA: Small range HL 1/11 pts			
Knoll et al. (74)	mTBI: M = 14, F = 38 Control: M = 24, F = 31	Mild (n52)	✓ (13)	✓ (20)	✓ (7)	✓ (4)	✓ (7)	✓ (1)	NR	**HHI-A:** Signif. higher in mTBI grp vs. control mTBI grp: Mostly severe scores in 43.8% HL was 2^nd^ reported symptom (n32) **Social/situational handicap (HHIA-S):** majority of mTBI grp (n14) **Emotional handicap (HHIA-E):** mTBI grp (n13)		Ti 3rd reported symptom in mTBI (n32) **THI score:** Signif. higher in mTBI grp vs. control mTBI: 21 pts. = Slight-mild score w/mTBI: 11 pts. = Moderate-catastrophic	Hy most frequent symptom in w/mTBI (n35) **HQ:** Signif. higher in mTBI grp vs. control w/mTBI: 9 pts. had clinically signif. Hy
										**Effect of aetiology** • No signif. difference in auditory symptoms across aetiology • **HHI-A:** No signif. difference in total mean scores by aetiology		**Effect of aetiology** • No signif. difference incl THI mean scores	**Effect of aetiology** • No signif. difference incl HQ mean scores
										**Effect of gender** • No signif. difference in number of auditory symptoms • **HHI-A**: No signif. difference in total mean scores		**Effect of gender** • No signif. difference • **THI scores:** No signif. difference in THI mean	**Effect of gender** • No signif. difference in number of symptoms • **HQ:** No signif. difference in HQ mean
Knoll et al. (82)	M = 10 F = 21	Mild (n22) Mod-severe (n9)	✓ (5)	✓ (13)	✓ (7)	✓ (2)	✓ (4)		NR	**PTA:** Normal (≤ 25 dB) in all **HHI-A:** Mild to severe scores in 71.4% pts. with HL &Ti		Ti 2nd reported symptom in 16 pts. **THI score**: 40% HL/Ti pts. reported mild -severe severity	Hy most reported symptom in 17 pts. **HQ:** 41.1% reported significant severity
										**Effect of severity** • mTBI grp: • HL was 3rd reported symptom (n11) • mean HHI-A score of 42.3(mild–moderate) • 21 (95.4%) reported ≥1 auditory symptom m-sTBI grp: • HL was 2nd reported symptom (n3) • mean HHI-A score of 26.6 (mild–moderate) • 7 (77.8%) reported ≥1 auditory symptom		**Effect of severity** mTBI grp: • Most reported symptom in 16 pts. • Mean THI of 17.1 (slight) m-sTBI grp: • Most reported symptom in 4 pts. • Mean THI of 28 (mild)	**Effect of severity** mTBI grp: • 2nd reported symptom in 14 pts. • Mean HQ of 26.8 (not clinically signif.) m-sTBI grp: • 2nd reported symptom in 3 pts. • Mean HQ of 20 (not clinically signif.)
Bunt et al. (94)	M = 52 F = 80	Concuss^	✓ NR	✓ NR	✓ NR	✓ NR		✓ NR	NR	“Adequate hearing to complete the interview & questions”			A medium effect size (Cohen’s d ≥ 0.50) of the symptom of noise sensitivity (0.55)
													**Effect of gender** • F reported greater symptom severity levels than M

ABLB, alternate binaural loudness balance test; ABR, auditory brainstem response; AC, Air conduction; ACs, auditory complaints; AR, acoustic reflex; BC, bone conduction; B/L, bilateral; CHL, conductive hearing loss; Concuss^, concussion; CST, competing sentences test; 1–3 pair DDR, 1-,2-,3- pair dichotic digit recognition; DDT, dichotic digits test; DL-FR, dichotic listening forced attention-right; DL-FL, dichotic listening forced attention; DL-NF, dichotic listening non-forced attention; DPOAE, distortion product otoacoustic emissions; DPT, duration patterns test; ECOG, electrocochleography; ESI, environmental sound identification test; Exam., examination; F, female; FPT, frequency patterns test; GIN, gaps-in-noise test; HFs, high frequencies; HHI-A, hearing handicap inventory for adults; HL, hearing loss; HQ, hypearcusis questionnaire; Hy, hypearacusis; IC, intearaural coherence; IPD, interaural phase difference task with 500 Hz stimuli; KDT, kana discrimination test; M, Male; MHL, mixed hearing loss; MLD, 500-Hz masking level difference; MLR/MLAEPs, middle latency response; MOSE, medial olivocochlear suppression effect; MRT, melody recognition test; m-sTBI, moderate–severe TBI; MT, monoaural testing; NRS (Loudness, discomfort, annoyance, ignorability and unpleasantness): Numeric Rating Scale; LE, left ear; LFs, low frequencies; LLR, late latency response; LS, Likert Scale; PTA, pure-tone audiometry; Pts, participants; Ouick-SIN, quick speech-in-noise test; RE, right ear; RPD, rhythm pattern discrimination; R-SPIN, revised speech perception in noise test; SA, speech audiometry; SCAN-3A, tests for auditory processing disorders in adolescents and adults; SDS, speech discrimination score; SL, sound localization; SRT_1_, speech reception threshold; SRT_2_, speech recognition threshold; SRM, spatial release from masking test; SRR, spectral ripple reversal detection; SNHL, sensorineural hearing loss; SSQHS, the speech, spatial and qualities of hearing scale; TCS, time compressed speech; TD, tone decay; TTD, two-tone discrimination test; TEOAE, transient evoked otoacoustic emissions; THI, tinnitus handicap inventory; TF, tuning fork; TFS, monoaural temporal fine structure perception; Ti, tinnitus; TM, tympanic membrane; TQ, tinnitus questionnaire; Tymp, tympanometry; WIN, words-in-noise test; WRS-Q, word recognition in quiet.

#### 3.3.1 Otoscopic assessment

In 27 (44%) studies, otoscopic assessment, a clinical procedure used to inspect the external auditory canal, tympanic membrane (eardrum), and middle ear (77), was conducted (11, 22, 23, 30, 35–37, 39, 44, 46, 51–53, 55, 56, 58, 59, 61–63, 65, 67, 70–73, 78). Some studies described otoscopic assessments as ENT, otologic, or clinical examinations (see Table 2). Eight studies presented clinical findings related to the tympanic membrane or external auditory canal without mentioning explicitly otoscopic assessment (e.g., intact eardrum) (25, 32, 38, 43, 54, 60, 64, 69) and 2 studies stated that otoscopy was performed, however the results were not reported (72, 73). In 18 (67%) out of the 27 records, the otoscopic assessment results or clinical findings indicated a normal eardrum (11, 22, 23, 25, 36, 37, 43, 44, 46, 53, 55, 56, 58, 63, 65, 70, 71, 78), whilst 16 (59%) studies noted at least one of the following symptoms: serous effusion, dried blood, blood, bloody otorrhea, cerebrospinal otorrhea, haemotympanum or haemorrhage (22, 30, 32, 35, 38, 39, 51, 52, 54, 59–62, 64, 67, 69). These symptoms were detected in the right ear in most of studies (10/16) (22, 30, 35, 38, 39, 52, 59–62).

#### 3.3.2 Pure-tone (behavioral) audiometry (PTA)

PTA refers to the assessment of thresholds determined by the lowest intensity at which an individual responds to sound at least 50% of the time (79). PTA was the most commonly used audiological assessment method with 56 studies reporting it (11, 22–43, 45–67, 69, 71–73, 75, 76, 78, 80–82). In four case studies, it was not explicitly stated whether PTA was conducted, but hearing loss was reported (49, 76), audiometer screening was performed (33) or audiometric findings were presented (31).

Normal hearing was reported for 19 (34%) out of 56 studies. Of these 19 studies, ten reported that hearing was normal or normal group mean bilaterally post-TBI (11, 33, 38, 41, 47, 59, 72, 73, 75, 82), whilst 9 studies reported normal hearing in at least one ear or in one case (22, 23, 26, 29, 39, 51, 56, 65, 80). Of these, two studies (11, 82), provided an accepted range for normal hearing (≤ 25 dB HL). The remaining 17 studies (17/19) provided no explanation, but nine (9/17) did demonstrate normal hearing with audiogram results of patients or groups mean thresholds (≤ 20 dB HL or 25 dB HL) (22, 26, 29, 39, 41, 47, 65, 72, 73).

Based on PTA assessment, the most commonly reported type of hearing loss post-TBI (n = 25, 45%) was SNHL (22, 23, 25–27, 36, 39, 42, 45, 46, 48, 50, 52, 53, 58, 61–67, 78, 80, 81). Among these, twelve (12/25) were identified as severe or profound SNHL (22, 26, 36, 39, 46, 58, 61–63, 65, 78, 81), with two case reports observing severe or profound SNHL in follow-up assessments (36, 46). Nine (9/25) were reported as mild or slight SNHL (22, 23, 42, 48, 64, 66, 67, 78, 81), with one case report observing mild SNHL in a follow-up assessment (67). In six studies (6/25), moderate SNHL was reported (27, 53, 63, 66, 67, 81), with one study noting this in a follow-up assessment (67). Following this, CHL (n = 12, 21%) was most reported (22, 24, 25, 30, 32, 35, 43, 44, 49, 54, 62, 64), whilst MHL (n = 7, 12.5%) was the least reported type of hearing loss (24, 26, 30, 39, 51, 60, 67). Three studies had no response to the stimulus in PTA assessment at all (37, 40, 55). In two other studies, no response was initially observed; however, SNHL was detected in the follow-up assessment before treatment (36, 46). In another study, MHL was observed in the initial PTA in left ear, however hearing worsened during follow-up, and no response was detected (39). In four studies, the type of hearing loss changed during follow-up assessments, and there were cases where hearing partially improved (22, 25, 30, 67). In ten studies, following PTA the degree (severity) of hearing loss or only hearing loss was reported, without reporting the type of hearing loss (in studies involving more than one case, at least one case) (22, 28–30, 34, 50, 56, 69, 71, 76). Eight out of ten studies reported severe to total (profound) hearing loss post-TBI (22, 28, 30, 34, 50, 56, 69, 76). One study stated that four frequencies (0.5 to 4 kilohertz (kHz)) were used to determine the average of hearing loss (71), whilst seven studies have not described the classification method used to determine the degree of hearing loss (i.e., mild, moderate and/or severe hearing loss) (22, 28, 30, 34, 50, 71, 76).

#### 3.3.3 Site-of-lesion tests

Site of lesions tests performed via audiometry are used to distinguish cochlear and retro-cochlear abnormalities (83). Four studies utilized 3 of the site-of-lesion tests (Békésy, Tone Decay and Alternate Binaural Loudness Balance (ABLB) test) (22, 28, 50, 66). Tone decay indicated findings in favor of retro-cochlear pathology in a patient with bilateral SNHL (66). Another study (50) that performed the Békésy test, reported a type I finding that indicated neither cochlear nor retro-cochlear pathology, even though the patient had bilateral SNHL. In one case study, the ABLB test showed no recruitment at low frequencies with severe hearing loss (28), whilst another study reported recruitment around 500 Hz in case 5 with SNHL in left ear (22).

#### 3.3.4 Tuning fork (TF) test (weber and/or Rinne)

The TF test is used for screening and determining the type of hearing loss, confirming PTA results (84, 85). Nine studies used the Rinne and/or Weber TF tests (22, 34, 39, 40, 46, 64–66, 70). In eight studies, the TF test results were consistent with the PTA results as seen in Table 2 (22, 34, 39, 40, 46, 64–66), whilst the remaining study did not perform PTA (70).

#### 3.3.5 Impedance audiometry (tympanometry and acoustic reflex thresholds)

Tympanometry objectively evaluates middle ear function (86, 87). The acoustic reflex thresholds (ART) assess auditory pathway integrity up to the superior olivary complex (SOC) via stapedius muscle reflex (88). In ten studies, both tympanometry and ART measurements were performed (11, 34, 37, 40, 43, 50, 54, 57, 67, 71), in eight only tympanometry was performed (36, 46, 55, 64, 66, 72, 73, 78) and in one study only ART measurement was conducted (39). Of the 18 studies (18/61) that performed tympanometry, normal (Type A) results were obtained from 12 (11, 34, 36, 37, 40, 46, 50, 55, 57, 71, 73, 78). The details of ART results performed ipsilaterally and/or contralaterally are presented in Table 2.

#### 3.3.6 Basic and advanced speech audiometry

Speech audiometry examines the ability to process speech in auditory centres, starting from the outer ear and ending with the cortex, using speech signals. Of 61 included records, both basic (e.g., speech reception threshold, speech discrimination score) and advanced (e.g., speech-in-noise tests) tests were performed in 2 (3%) studies (11, 73), while basic speech audiometry test(s) were performed in 13 (21%) studies (26, 32, 37, 39, 47, 50, 53, 56, 57, 63, 66, 71, 78), including six studies using Speech Discrimination Score (SDS) (26, 32, 50, 57, 66, 71), four using Speech Reception Threshold (11, 37, 53, 73) and four using Speech Recognition Threshold (11, 32, 47, 73). The results of the tests varied depending on the patients or cases, from normal to no response at all (Table 2). A common result was not identified. Studies with follow-up assessments reported improvement in SDS results over time for one case with bilateral mild to moderate hearing loss (71), whilst another reporting worsening of SDS in one case with bilateral SNHL (50). More advanced, QuickSIN test was used in two studies (11, 73), Words-in-Noise (WIN) test was used in one of those studies (73). In both studies, although the average hearing was normal post-TBI, mild signal-to-noise-ratio (SNR) loss or an abnormal result in at least one ear was observed in the QuickSIN results. Similarly, in the WIN test, abnormal results were reported in at least one ear across 8 participants (73).

#### 3.3.7 Otoacoustic emissions (OAEs) and suppression test

OAEs provide an objective assessment of the functionality of the outer hair cells in the cochlea (89). Only 6 (10%) studies out of the 61 records used OAEs. In 2 studies, both Distortion Product OAE (DPOAE) and Transient Evoked OAE (TEOAE) measurements were employed (37, 71), in three DPOAE was measured (47, 50, 66), and one study measured TEOAE (72). In studies where only DPOAE was performed, the DPOAE was obtained in normal hearing (47), whilst it was absent or very poor in cases of SNHL (50, 66), consistent with the hearing conditions of patients. In one case study, bilateral responses were observed in both TEOAE and DPOAE (up to 3 kHz or 4 kHz) in a patient with mild hearing loss (71), whilst another case study observed bilateral responses of TEOAE and DPOAE (only absent at 2 kHz) despite no response being obtained in either PTA or the ipsi-contralateral ART (37). In a study comparing a control group to a TBI group with/without auditory complaints (e.g., tinnitus, difficulty of hearing in noise, hyperacusis), where hearing was within normal limits in all groups, it was observed that the TEOAE amplitudes of the entire TBI group were lower than those of the control group. However, the amplitudes of the TBI group with auditory complaints were higher than those without auditory complaints (72). In one study, an OAEs suppression test referred to as medial olivocochlear suppression effect (MOSE) test, which allows for the evaluation of the efferent system (90), indicated that an absent effect of the auditory efferent system in one or both ears of the TBI patients with auditory complaints (72).

#### 3.3.8 Electrophysiological tests

Electrophysiological tests performed with auditory potentials enable the evaluation of the auditory pathway from the auditory nerve to more central regions in the brain (91). Out of the 61 records, 16 (26%) studies used electrophysiological tests, with 15 studies using Auditory Brainstem Response (ABR/BAEP) (15/16) (34, 36, 37, 40, 41, 46, 47, 50, 53, 55, 58, 66, 68, 71, 80), one study using electrocochleography (ECOG) (38) and five using additional tests, including middle latency responses (MLR/MLAEPs) (41, 47), late latency responses (LLR) (66, 71), and mismatch negativity (MMN) and P300 (66).

Out of the 15 studies, 13 showed that ABR findings were consistent with PTA results (34, 36, 37, 41, 46, 47, 50, 53, 55, 58, 66, 71, 80). For instance, in cases of bilateral profound SNHL, either bilateral unobtainable ABRs were observed (58) or, depending on the degree of hearing loss, waves I and III were obtained, but no peak in wave V was observed (66). In cases with normal hearing normal ABR results (47), or prolonged latency in wave V were obtained (41). However, in one of these studies, ABR, PTA and ART results were not obtained consistently, whilst results of OAEs were present (37) (refer to the OAEs section). In one study, ABR results were in a normal waveform and no response was obtained in PTA, whilst ART results were present bilaterally (40). Another study by Shibata (68) reported cortical deafness due to delayed traumatic intracerebral haematoma using Magnetic Resonance Imaging (MRI), but 1 month later a normal response was observed in the ABR performed (68). Furthermore, in two case studies (50, 71), improvement in ABR results in follow-up assessments corresponded to improvement in the degree of hearing loss obtained in PTA (71), whilst deterioration in ABR results corresponded to worsening in the degree of hearing loss (50). The details of other electrophysiological test results are presented in Table 2.

#### 3.3.9 Central auditory tests

Central auditory system assessments facilitate the evaluation of auditory processes such as the processing, interpretation, and discrimination, enabling the assessment of the central levels of the auditory pathway (92). Out of 61 records, 5 (8%) studies performed various central auditory tests (11, 33, 41, 47, 73), despite normal hearing reported in PTA, abnormal results were observed in at least one central auditory test (Table 2). The age of participants in these studies ranged from 22 to 71 years.

#### 3.3.10 PROMs

PROMs (93) were used in 8 (13%) out of the 61 studies (11, 57, 71, 73, 74, 76, 82, 94) for assessment of hearing (11, 71, 73, 74, 82), tinnitus (57, 71, 74, 76, 82) and hyperacusis (11, 74, 82). PROMs used include Hearing Handicap Inventory for Adults (HHI-A) (11, 71, 74, 82), the speech spatial and qualities of hearing scale (73), the screening checklist for Auditory Processing in Adults (71), Tinnitus Handicap Inventory (THI) (71, 74, 82), a Likert scale for tinnitus amplitude (76), the Tinnitus Questionnaire (TQ) and numeric rating scale (NRS) for loudness, discomfort, annoyance, ignorability, and unpleasantness (57) and Hyperacusis Questionnaire (HQ) (11, 74, 82) were performed. In one study, the post-concussion symptom scale (PCSS) was used (94). In most studies (5/8), more than one PROM was used (11, 57, 71, 74, 82).

Hearing impairment was observed in all studies in which HHI-A was reported (11, 71, 74, 82). In particular, although normal hearing was detected in PTA in two studies, mild to severe (82) or substantial impairment (11) was observed because of HHI-A. Similarly, for the 16 (26%) studies reporting complaints of tinnitus (11, 24, 26, 28–30, 39, 47, 51, 57, 59, 61, 71, 72, 76, 78), in the 3 studies using THI a range from slight to catastrophic score was reported (71, 74, 82). In the case study where TQ and NRS were used (57), the tinnitus severity grade was reported as extreme, and the patient considered tinnitus to be a very big problem in the NRS. Furthermore, tinnitus was reported to worsen from the time of TBI to initial consultation. Three (5%) studies reported complaints of hyperacusis (11, 60, 72), out of which 2 studies found that hyperacusis was the most reported symptom among individuals with TBI using HQ. Both studies reported significant sensitivity based on HQ results (74, 82). In the case study where HQ was used, the patient found all sounds too loud and reported substantial impairment (11). Hyperacusis was also reported in the study using PCSS (94). Detailed results of other PROMs are presented in Table 2.

### 3.4 Effect of severity of non-blast related TBI on auditory outcomes

The majority of studies have not clearly stated the severity of TBI (38/61) (22–30, 35, 36, 38–45, 48, 50–56, 60–62, 65–70, 76, 81). Of the remaining, 10 studies included severe TBI (24, 30, 33, 37, 46, 57, 58, 64, 71, 80), 6 included mild TBI (11, 47, 63, 73, 74, 78), 7 studies reported concussion (i.e., mild TBI) (29, 31, 32, 59, 75, 81, 94), 2 observed moderate/severe TBI (34, 49) and 2 included a range from mild to severe TBI patients (72, 82) (see Table 1 more details on severity, e.g., criteria of severity).

In the 2 studies with a range of mild to severe TBI (72, 82), normal hearing (≤ 20 dB HL or 25 dB HL) was observed and tinnitus and/or hyperacusis were reported. In Knoll et al. (82), tinnitus was the commonly reported symptom in both mild-TBI and moderate–severe-TBI groups. However, the mean for THI was higher in the moderate–severe-TBI group indicating more severe score than the mild TBI group (Table 2).

In 6 studies where TBI severity was classified only as mild (11, 47, 63, 73, 74, 78), abnormal results were observed in at least one central auditory test despite normal hearing in three studies (11, 47, 73), the remaining studies did not perform central hearing tests (63, 78, 82). In one study for mild TBI, a severe hearing impairment was reported using HHI-A (74). In patients exposed to mild TBI, Jang, Bae and Seo (63) observed moderate and severe SNHL, whilst Jafarzadeh et al. (78) reported mild to profound SNHL. Tinnitus was observed in four studies involving mild TBI (11, 47, 74, 78), whilst two studies reported both hyperacusis and tinnitus (11, 74). HQ results of these studies are explained in the PROMs section earlier. In the remaining three studies (11, 47, 78), tinnitus was reported; however, no formal assessment was conducted. Notably, one of these studies, the reported tinnitus resolved a several months later (47). Also, different outcomes were observed in each of the studies reporting concussion such as normal hearing, mild CHL and profound SNHL (29, 31, 32, 59, 75, 81). Hyperacusis was observed after concussion (94), and complaint of tinnitus were reported in another study (59).

**Figure 2:**
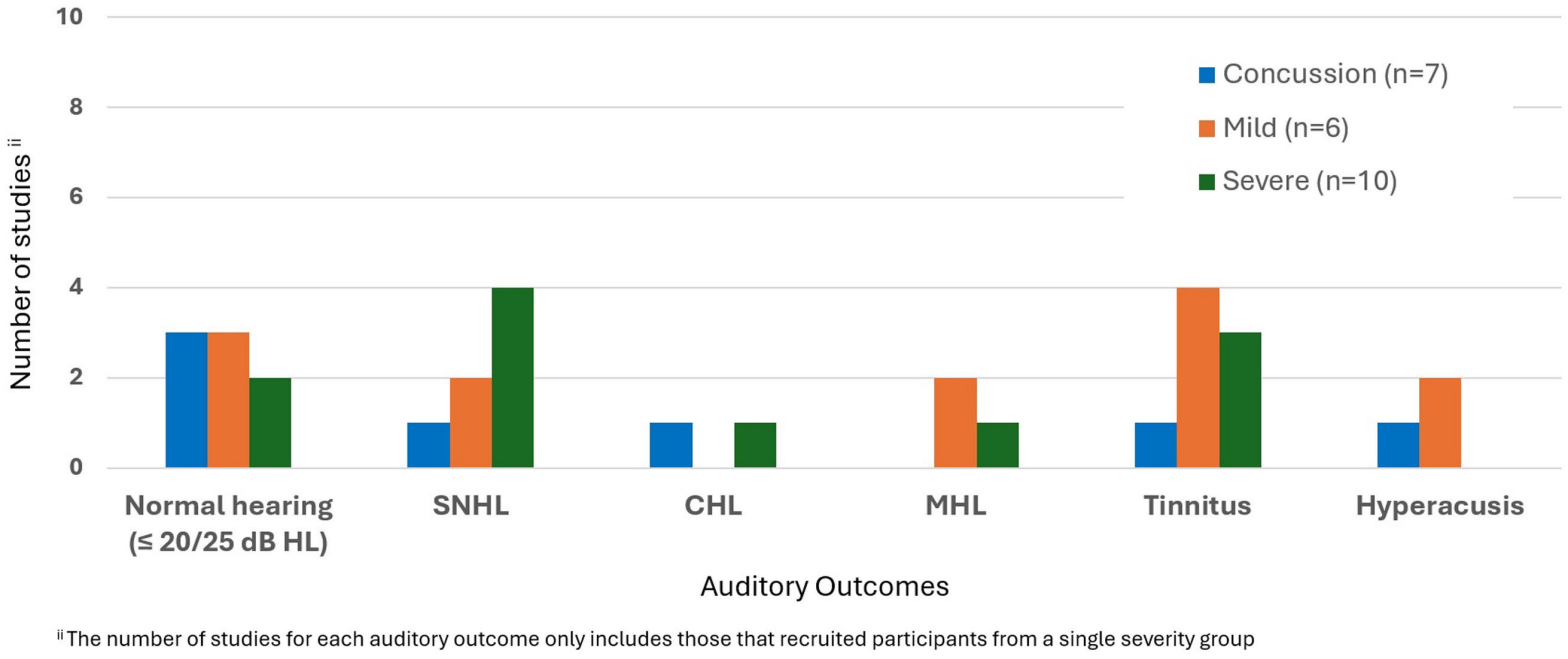
Distribution of auditory outcomes based on severity of non-blast related TBI.

SNHL was reported in 4 out of 10 studies reporting severe TBI (46, 58, 64, 80). This group also exhibited a range of outcomes from normal hearing to total deafness as well as CHL. There were tinnitus complaints in three case studies in severe TBI (30, 57, 71). In 2 studies evaluating tinnitus in this group, catastrophic score was detected in THI for mild hearing loss (71), and extreme tinnitus severity was observed in TQ in normal hearing between 0.125–2 kHz, with a steep decline toward higher frequencies on both sides (57). Hyperacusis was not indicated in any of the studies that included only severe TBI. In four of those studies, abnormal results were observed in at least one component of ABR (e.g., wave V) at the brainstem level, despite normal hearing or varying types or degrees of hearing loss (37, 46, 58, 71). Figure 2 shows the distribution of auditory outcomes across studies according to TBI severity.

In summary, the severity of TBI may not consistently predict auditory outcomes and both mild and severe TBI can result in significant auditory impairments and abnormal central auditory test results.

### 3.5 Effect of aetiology of non-blast related TBI on auditory outcomes

In terms of aetiology, the majority of studies (36/61) reported motor vehicle accidents (MVA) at least one participant or case (Table 1) (11, 22–25, 28–30, 32–34, 37, 38, 40, 42, 43, 49, 53, 54, 58, 60, 62–64, 66, 67, 69, 71–74, 76, 80–82, 94).

To examine the effect of aetiologies related to TBI, they were classified into five categories: MVA, falls, sports-related injuries, assaults, and others. In six studies involving multiple participants, different aetiologies, from MVA to assault, were included (72–74, 80, 82, 94). For these studies results are reported together under all aetiologies. In one of the 6 studies (73) the group mean showed normal hearing, but a small range of hearing loss was reported in at least one ear in three participants with TBI. However, the degree classification of hearing loss was not explained (Table 2) (73). For these participants, two had an aetiology of fall, whilst one was due to MVA. In another study by Knoll et al. (74), it was observed that there was no significant difference in the presence of auditory symptoms across aetiology of the TBI. For the remaining 3 studies, two studies reported normal hearing for all participants (72, 82), whilst the other study reported SNHL for only one participant, but the aetiology was not specified (80).

Out of the 30 studies reporting MVA in case series/studies, 12 studies reported SNHL in at least one case and/or ear (22, 23, 25, 42, 53, 58, 62–64, 66, 67, 81), 10 studies reported CHL (22, 24, 25, 30, 32, 43, 49, 54, 62, 64), 4 studies reported MHL (24, 30, 60, 67), although two studies reported that the type of hearing loss changed in follow-up assessments (30, 67) and 6 studies reported normal hearing in at least one case and/or ear (11, 22, 23, 29, 33, 38). However, in two of these studies, despite normal hearing, abnormal results were obtained in at least one central auditory test, leading to diagnoses of auditory attentional neglect (33) or auditory processing deficits (11). Five studies reported tinnitus complaints following MVA (11, 28, 30, 71, 76), and one case reported hyperacusis linked to MVA (60).

Across studies reporting falls (21/61) in case series/studies, SNHL was observed in 10 studies (22, 26, 36, 45, 46, 48, 50, 65, 78, 81), followed by normal hearing in 4 studies (23, 47, 51, 65), MHL across 3 studies (26, 39, 51), and CHL in one study (32) at least one ear and/or one case. In 6 studies, either hearing loss without the type was reported or a diagnosis (e.g., unilateral ossicular chain disruption) was noted (29, 31, 55, 57, 68, 70). Five studies report tinnitus complaints following falls (26, 29, 47, 51, 57), and one study reported hyperacusis (11).

**Figure 3:**
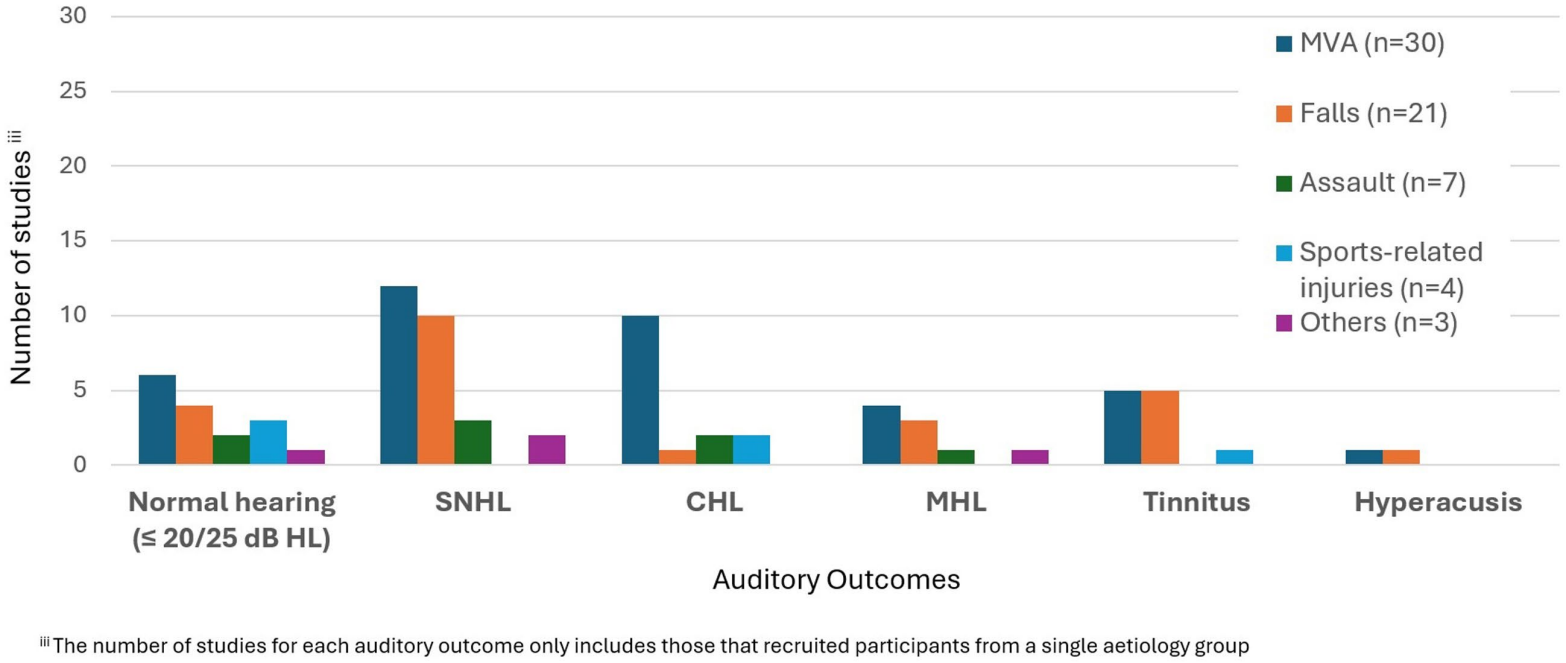
Distribution of auditory outcomes based on aetiology of non-blast related TBI.

Out of 7 studies reporting different types of assaults in case series/studies, normal hearing (39, 56), and all types of hearing loss [SNHL (26, 39, 61)], [CHL (30, 44)], and [MHL (24)] in at least one ear and/or one case, and tinnitus in (24, 26, 39, 61) were observed. In the 4 studies reporting sports-related TBI (35, 41, 59, 75), normal hearing or normal hearing with a brainstem auditory-processing disorder were observed across three studies (41, 59, 75), CHL was observed in one case study (35) and tinnitus was reported in one study (59). Three studies were categorised under ‘other’ aetiologies: striking the back of the head (39), industrial injury (52), and an object falling from a bookcase (27). In two of these studies, SNHL was detected (27, 52), whilst Lyos et al. (39) initially observed MHL, one week later, one ear had normal hearing, and no response was obtained in PTA in the other. Figure 3 illustrates the distribution of auditory outcomes according to the aetiology of non-blast related TBI.

Similar to TBI severity, various auditory outcomes ranging from normal hearing to different types of hearing loss were observed across aetiologies of TBI. Additionally, tinnitus and hyperacusis were reported across different aetiologies.

### 3.6 Effect of gender on auditory outcomes following non-blast related TBI

In terms of gender, out of 33 studies that included only male patients (n of male = 43) (22, 24, 26, 28, 31, 33, 35, 36, 39, 41, 42, 44–46, 48–50, 52, 55–62, 64–68, 71, 76), SNHL was reported in 17 studies in a total of 21 male (22, 26, 36, 39, 42, 45, 46, 48, 50, 52, 58, 61, 62, 64–67). Normal hearing in 8 studies, comprising 9 male cases (22, 26, 33, 39, 41, 56, 59, 65), CHL across 7 studies in 8 males (22, 24, 35, 44, 49, 62, 64), and MHL in 5 studies at least one ear and/or one case in six males (24, 26, 39, 60, 67). Tinnitus complaints were reported in 9 of these studies, in a total of 11 male (24, 26, 28, 39, 57, 59, 61, 71, 76), whilst only one case study noted hyperacusis before the assessment (60). The results for other male patients are detailed in Table 2.

**Figure 4:**
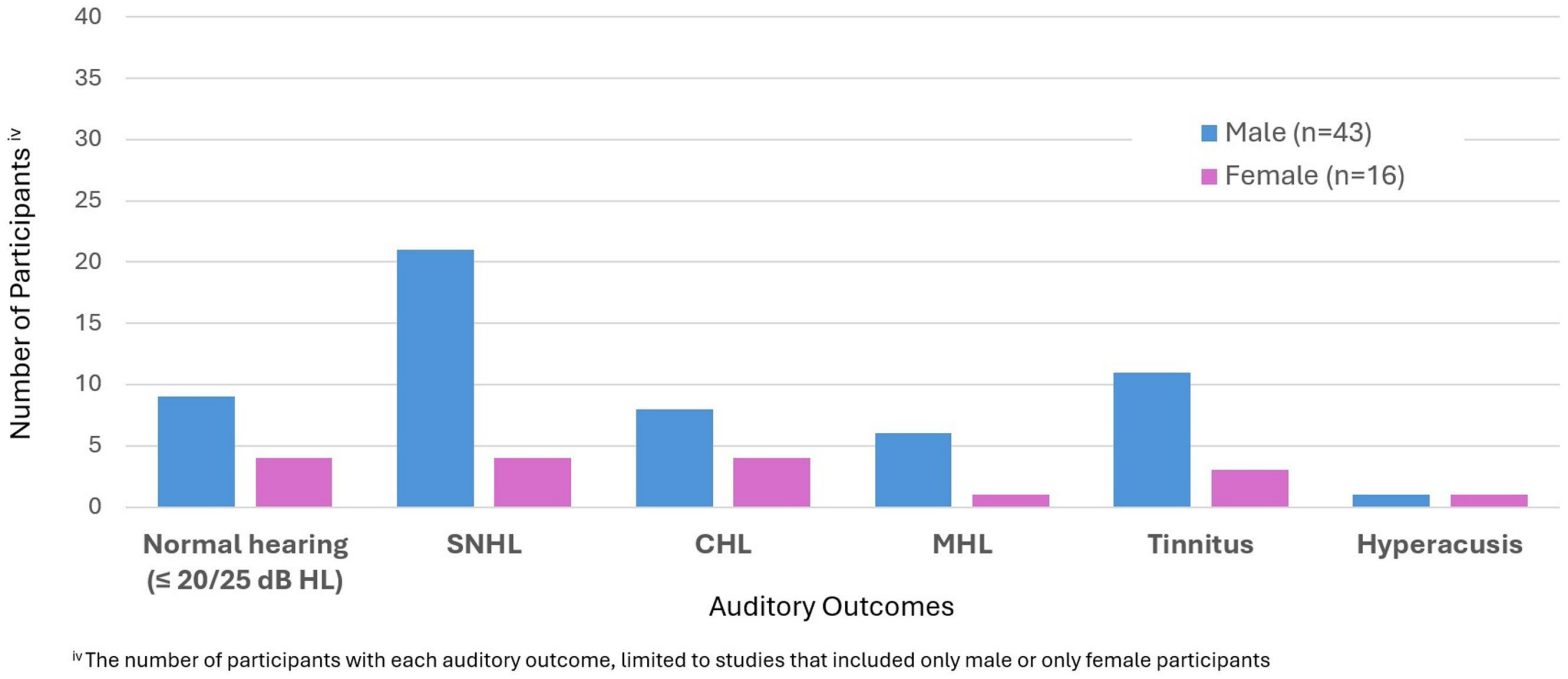
Distribution of auditory outcomes by gender following non-blast related TBI.

In 15 studies involving only female participants (*n* of females = 16) (11, 25, 27, 34, 37, 38, 40, 43, 47, 51, 53, 54, 63, 69, 70), normal hearing was reported across 4 studies and in 4 females (11, 38, 47, 51), SNHL in 4 case studies (25, 27, 53, 63), CHL in 3 studies and in 4 females (25, 43, 54), and MHL in one case study (51). There were also female cases where no response was obtained in PTA (37, 40), or total hearing loss was observed (69). Additionally, tinnitus complaints before assessment were noted in three studies and females (11, 47, 51), with one study reporting hyperacusis in addition to tinnitus (11). The distribution of auditory outcomes from studies that included only male or only female participants was shown in Figure 4.

When cases with the same aetiology and severity (e.g., MVA-related severe or mild TBI) were compared within each gender, auditory outcomes still varied from normal hearing with abnormal central auditory tests (11, 33) to moderate-to-severe hearing loss, including SNHL (58, 63, 64) in both males and females.

Of the 12 studies that included both genders (23, 29, 30, 32, 72, 74, 75, 78, 80–82, 94), 4 studies included more males than females (72, 75, 78, 80), and 3 studies included more females than males (74, 82, 94). Seven studies involved multiple participants (72, 74, 75, 78, 80, 82, 94), of which four studies reported normal hearing or a mean of normal hearing (72, 75, 80, 82), except for one participant with SNHL (gender not specified) (80). Another study with 20 males and one female reported SNHL in 47.6% of participants (78). In the five case studies involving both genders (23, 29, 30, 32, 81), hearing conditions ranged from normal hearing (3 females) to CHL (1 female, 3 males) and SNHL (1 female, 4 males). No MHL was reported in female cases, whilst the MHL reported in one male (Case 3) later turned into CHL (30).

Six studies reported participants experiencing tinnitus and hyperacusis. In two case studies, participants reporting tinnitus were male (29, 30). In another cross-sectional study, the number of males with auditory complaints including tinnitus and/or hyperacusis was higher than females, however no formal statistical analysis was undertaken (72). In one study, no significant differences were found between genders in THI and HQ mean scores (74), whereas another study reported that females had greater symptom severity levels than males in the PCSS in relation to hyperacusis (94). Finally, Jafarzadeh et al. (78) did not report the gender of the participants reporting tinnitus.

Overall, the studies showed a range of auditory outcomes based on gender. SNHL was frequently reported in studies with male participants (17/33, 52%), whilst normal hearing and other types of hearing loss were noted in both genders. Tinnitus and hyperacusis were observed in both males and females.

## 4 Discussion

This scoping review compiled the common auditory impairments of non-blast related TBI, along with exploring the impact of severity, aetiology of TBI, and gender on auditory outcomes. The predominance of case studies compared to other research designs makes it difficult to generalize the results due to individual differences.

In terms of assessment, PTA was the most commonly used assessment method, followed by otoscopic assessment; in contrast, other audiological assessments (e.g., OAEs, central auditory tests and electrophysiological measures) were applied in less than 30% of studies. Inconsistencies in the assessment methods employed indicate a lack of both methodological and clinical standardization in studies conducted in this field. Furthermore, audiological training emphasises the need for performing tests based on a holistic approach and the principle of cross-checking (95), whilst the differences among records can suggest that this approach is not strictly adhered to in practice. However, the presence of abnormal results in central auditory tests (11, 33, 41, 47, 73) or the observation of auditory symptoms such as tinnitus, hyperacusis and difficulty understanding speech-in-noise (11, 72, 73, 82) even in individuals with normal hearing post-TBI, underscores the importance of auditory assessments ranging from PROMs to central auditory tests in this patient group. For instance, in a case study, Cevette et al. (37) observed bilateral results in TEOAE and DPOAE, which indicated normal outer hair cell function, even though results in ipsi-contralateral ARTs and abnormal ABR findings at 90 dB normalized hearing-level (nHL) and as such highlighted the importance of applying OAE tests when investigating potential involvement of different auditory pathway regions due to non-blast TBI. In addition, although the included studies comprehensively assessed the auditory pathway through various tests, as shown in Supplementary Appendix Table 4, some assessments, such as extended-high frequency audiometry (EHFs) and uncomfortable loudness levels (ULLs), were not performed in any of the studies. These assessments may be important for this patient group, or if they are not applied, the reasons for their non-application should be justified. These findings further support the argument for standardising post-TBI audiological assessments, particularly in light of the variability in test application despite the presence of significant auditory symptoms.

Consistent with previous literature (14, 96), the most common type of hearing loss following non-blast related TBI was SNHL (*n* = 25). Nevertheless, drawing any definitive conclusions can be difficult due to the observation of both normal hearing and other types of hearing loss. Across all studies that performed PTA, the lack of reporting of the type of hearing loss, the accepted classification method for degree of hearing loss, and/or frequencies used to calculate the pure-tone averages also hinder reaching general conclusions about hearing loss associated with non-blast related TBI. Significantly, cases where the type and degree of hearing loss, and/or auditory symptoms change over time (22, 25, 30, 36, 39, 46, 50, 71) show the importance of refraining from making a definitive diagnosis at the initial assessment following non-blast related TBI and emphasize the necessity for regular follow-up assessments in this patient group. Future research is needed with large sample sizes to determine the ideal/recommended time points for audiological assessment post-injury.

Although patients complained of tinnitus and/or hyperacusis, neither PROMs nor any specific methods were used to assess these symptoms across all studies (24, 26, 28–30, 39, 51, 57, 59–61, 72, 78). This finding may suggest that there were no recommended guidelines for earlier studies or that existing guidelines are not universally/commonly adopted at present, indicating a lack of standardization in assessment (97–100). The THI and HQ are among the most commonly used PROMs in the UK (101, 102), and our results of studies using PROMs aligned with this (11, 71, 74, 82). In our review, studies reported a range of tinnitus severity related to TBI, from slight to catastrophic. This could indicate the diverse impacts of TBI on each patient. The fact that hyperacusis is the most commonly reported symptom among TBI patients in studies using the HQ (74, 82) highlights the importance of not overlooking hyperacusis in these patients. Therefore, it is essential to have standardized practices for the assessment of tinnitus and/or hyperacusis in this patient population.

Furthermore, this review highlights the limited use of PROMs across auditory complaints, despite patient-reported symptoms. The limited reports of PROMs may reflect a global lack of awareness or willingness to use PROMS in clinical and/or research contexts and the inclination to prioritise traditional audiological assessments, such as PTA. Another potential reason for limited use is the lack of language-specific validated PROMs for non-English-speaking countries. Whilst traditional audiological assessments do provide essential assessment information, PROMs provide a better understanding of the individual effects of the symptoms which inform both the diagnostic process and intervention plans in a holistic manner (103). Moreover, PROMs are important to evaluate the impact and effectiveness of management strategies on patients’ well-being, functional status and psychosocial needs (104).

In terms of severity, the presence of similar auditory symptoms and types of hearing loss across different severities of TBI suggests that auditory outcomes may arise independently of TBI severity. However, the absence of a study specifically evaluating moderate TBI, inconsistent reporting of TBI severity across studies, and the existing literature indicating a correlation between TBI severity and hearing loss (105, 106) prevent a definitive conclusion on this matter. Furthermore, the lack of consistent reporting of severity criteria among studies that specified TBI severity, and the use of different criteria (e.g., GCS, DSM-5) in the few studies that did report them, make it difficult to draw robust and generalisable conclusions about the impact of TBI severity on auditory outcomes. Although the widely used GCS classification system was introduced in 1974 (6), the earliest study among those included that reported TBI severity was published in 1984 (30), and this study did not specify the criteria used. The earliest study in our records that reported both severity and the criteria for determining it dates back to 2005 (72). This highlights how historical changes in definitions and classifications may affect data comparability. Therefore, future studies should consistently report both the TBI severity and the criteria used for its determination.

Similarly, the observation of normal hearing, all types of hearing loss, and tinnitus in MVAs, falls, and assaults, suggests that aetiology may not have a specific effect on auditory outcomes. Therefore, no definitive framework can be drawn for symptoms related to aetiology. Notably, studies related to sports injuries did not report SNHL and MHL, however, this finding is not sufficient for generalization. Further studies are needed to evaluate the impact of TBI aetiology on auditory outcomes.

The predominance of males who experienced TBI can likely be attributed to the higher incidence of TBI among males, as observed in epidemiological studies (107, 108). Auditory symptoms such as tinnitus and hyperacusis were observed in both genders. SNHL was observed more frequently in male patients, whilst there were no notable differences observed for female patients in the type of hearing loss. Even when similarities in severity and aetiology were controlled, there was still range in auditory outcomes for both genders. However, it should be noted that the imbalance in gender distribution may affect the overall validity of this finding. In the similarity comparison conducted to minimize bias arising from gender imbalance, the presence of different auditory outcomes across both genders impeded clear gender-based interpretations.

The main focus of this review was not to investigate age-related effects of TBI; however, the age range of participants in the studies (from young to older adults) raises important conditions. For instance, in several cases, despite normal hearing, abnormal central auditory test results were observed even in younger adults, which can be considered an important finding for more clearly tracking the direct effects of TBI. However, in studies that include middle-aged and older adults, the potential contribution of age-related central auditory processing decline or hearing loss should not be overlooked (109). Moreover, particular age groups are at higher or lower risk of TBI (110). It is also recognized that neural plasticity varies across the lifespan, which may influence the brain’s response to injury (111). These findings highlight the necessity of considering age-related comparisons when interpreting auditory outcomes in future studies of the TBI population, as age can act as a compound factor affecting both peripheral and central auditory functions.

Despite the older studies dating back to 1956 in this field, the complex nature of TBI and the lack of a guideline and/or standardization in auditory assessment within this patient group make it challenging to establish a comprehensive framework for auditory outcomes. Current findings indicate a wide variation in auditory outcomes based on TBI severity, aetiology and gender. This underscores the need for standardization in assessment and reporting, particularly within the TBI patient group, beginning from general audiological assessments. For this purpose, a guideline should be developed for assessing auditory outcomes in non-blast related TBI patients, and the effect of TBI variables on outcomes should be investigated through larger, systematic research designs in future studies.

### 4.1 Strengths and limitations

This scoping review provided a comprehensive evaluation of the research objectives through an extensive literature review and analysis. The investigation of the potential effects of TBI severity, aetiology and gender variables on auditory outcomes allowed for an in-depth analysis and insights into the impact of these factors on auditory conditions. However, although the assessment time of the auditory outcomes related to TBI was reported throughout the records, potential differences in auditory outcomes due to assessment time were not examined within this review. Future studies should consider exploring the impact of assessment time on auditory outcomes. In addition, an imbalance in the sample representation of gender, such as a predominance of male participants, limited the generalizability of the findings related to the effects of this variable on auditory outcomes. By conducting a detailed review of studies containing terms such as head injury, fracture, and thalamic lesion, we ensured that only those meeting the diagnostic criteria of TBI (described in inclusion criteria) were included. This allowed us to directly report the auditory consequences of non-blast related TBI. However, it should be recognized that this review only included studies published in English and as such the findings may not be as generalizable to other non-English speaking countries, although studies were included from a range of countries.

## 5 Conclusion

The compiled findings highlight the diversity of auditory outcomes associated with non-blast related TBI. However, the lack of standardization in audiological assessment methods and reporting, not conducting further assessments (e.g., central auditory tests) in cases of normal hearing, and/or not frequently assessing other audiological symptoms such as tinnitus and hyperacusis hinder a definitive conclusion about the auditory outcomes of TBI patients. Furthermore, these can complicate the diagnosis and treatment process, leading to worsening auditory conditions in TBI patients. All these audiological deficiencies also negatively affect the determination of the effect of variables such as TBI severity, aetiology and gender on auditory outcomes. Therefore, it is crucial to determine standard audiological practices for assessing, reporting, and managing auditory conditions in TBI patients. Following the establishment of these standards, there is a need for specifically designed large-sample size studies with more balanced sample characteristics (e.g., gender or aetiology) to determine the effects of variables on auditory outcomes of non-blast related TBI patients.

## Abbreviations

ABLB: Alternate Binaural Loudness Balance
ABR: Auditory Brainstem Response
ART: Acoustic Reflex Thresholds
CHL: Conductive Hearing Loss
DPOAE: Distortion Product OAE
ECOG: Electrocochleography
EHFs: Extended-high Frequency
ENT: Ear, Nose and Throat
HHI-A: Hearing Handicap Inventory for Adults
HQ: Hyperacusis Questionnaire
GCS: Glasgow Coma Scale
kHz: Kilohertz
LLR: Late Latency Responses
MHL: Mixed Hearing Loss
MLR: Middle Latency Responses
MMN: Mismatch Negativity
MOSE: Medial Olivocochlear Suppression Effect
MRI: Magnetic Resonance Imaging
MVA: Motor Vehicle Accident
nHL: Normalized hearing-level
NRS: Numeric Rating Scale
OAEs: Otoacoustic Emissions
PCSS: Post-Concussion Symptom Scale
PROMs: Patient-Reported Outcome Measurements
PTA: Pure tone Audiometry
SDS: Speech Discrimination Score
SNHL: Sensorineural Hearing Loss
SNR: Signal-to-Noise-Ratio
SOC: Superior Olivary Complex
TBI: Traumatic Brain Injury
TEOAE: Transient Evoked OAE
TF: Tuning Fork
THI: Tinnitus Handicap Inventory
TQ: Tinnitus Questionnaire
ULLs: Uncomfortable Loudness Levels
WIN: Words-in-Noise
