# Correction: The auditory outcomes in non-blast related traumatic brain injury and the role of severity, aetiology and gender: a scoping review

**DOI:** 10.3389/fneur.2025.1666664

**Published:** 2025-08-12

**Authors:** Kübra Bölükbaş, Laura Edwards, Olivia R. Phillips, Kathryn Fackrell

**Affiliations:** ^1^Hearing Sciences, Division of Mental Health and Clinical Neuroscience, School of Medicine, University of Nottingham, Nottingham, United Kingdom; ^2^National Institute of Health and Social Research (NIHR) Nottingham Biomedical Research Centre, Nottingham, United Kingdom; ^3^Division of Rehabilitation Medicine, University Hospitals of Derby and Burton NHS Foundation Trust, Derby, United Kingdom; ^4^Centre for Rehabilitation and Ageing Research, School of Medicine, University of Nottingham, Nottingham, United Kingdom; ^5^Lifespan and Population Health, School of Medicine, University of Nottingham, Nottingham, United Kingdom

**Keywords:** traumatic brain injury, auditory, hearing loss, tinnitus, hyperacusis, TBI severity, aetiology, gender

In the published article an error was identified regarding the reference numbers in Table 1 as published. The old version appears below:

**Figure:**
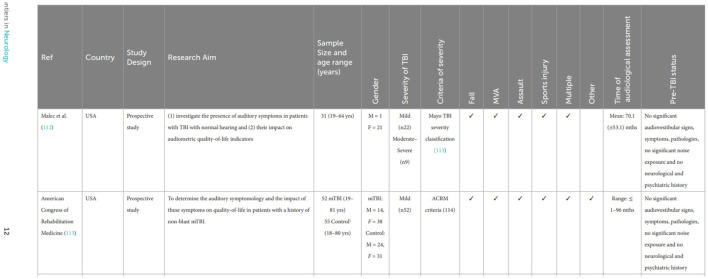


As shown in the Table above, in the first and second lines, the studies by Knoll et al. (82) and Knoll et al. (74) were mistakenly replaced with the references of the severity classification systems (Malec et al. and the American Congress of Rehabilitation Medicine) in the author column. The updated version appears below:

**Figure:**
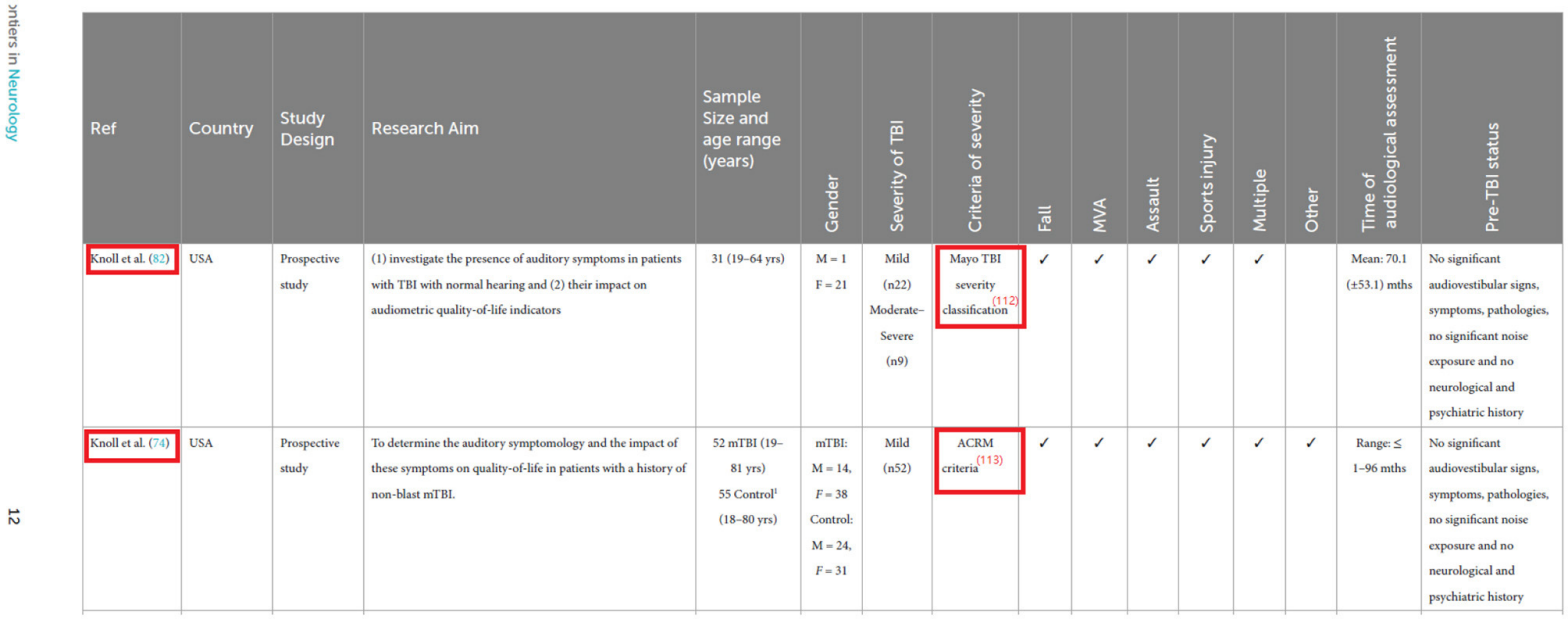


As highlighted in red, the first row should list Knoll et al. (82) as the study. The reference in the “Criteria of severity” column should be “Mayo TBI severity classification” with the reference number written inside the parenthesis: Mayo TBI severity classification (112). The second row should list Knoll et al. (74) as the study. The reference in the “Criteria of severity” column should be “ACRM criteria” with the reference number written inside the parenthesis: ACRM criteria (113).

The original version of this article has been updated.

