# Supplementary material for: The auditory outcomes in non-blast related traumatic brain injury and the role of severity, aetiology and gender: a scoping review
Source: Front Neurol. 2025 Jul 10;16:1589117. doi: 10.3389/fneur.2025.1589117 (PMC12288686; doi:10.3389/fneur.2025.1589117)
Supplement: Supplementary file 1 [file Data_Sheet_1.pdf]

Supplemental Appendix

Table 1. Preferred Reporting Items for Systematic reviews and Meta-Analyses extension for Scoping Reviews (PRISMA-ScR) Checklist

SECTION	ITEM	PRISMA-ScR CHECKLIST ITEM	REPORTED IN SECTION OR PAGE #
**TITLE**			
Title	1	Identify the report as a scoping review.	#1
**ABSTRACT**			
Structured summary	2	Provide a structured summary that includes (as applicable): background, objectives, eligibility criteria, sources of evidence, charting methods, results, and conclusions that relate to the review questions and objectives.	#Abstract
**INTRODUCTION**			
Rationale	3	Describe the rationale for the review in the context of what is already known. Explain why the review questions/objectives lend themselves to a scoping review approach.	#Introduction
Objectives	4	Provide an explicit statement of the questions and objectives being addressed with reference to their key elements (e.g., population or participants, concepts, and context) or other relevant key elements used to conceptualize the review questions and/or objectives.	#End of Introduction
**METHODS**			
Protocol and registration	5	Indicate whether a review protocol exists; state if and where it can be accessed (e.g., a Web address); and if available, provide registration information, including the registration number.	NA
Eligibility criteria	6	Specify characteristics of the sources of evidence used as eligibility criteria (e.g., years considered, language, and publication status), and provide a rationale.	#Identifying relevant studies-Eligibility criteria
Information sources*	7	Describe all information sources in the search (e.g., databases with dates of coverage and contact with authors to identify additional sources), as well as the date the most recent search was executed.	#Seach strategy
Search	8	Present the full electronic search strategy for at least 1 database, including any limits used, such that it could be repeated.	#Search strategy, Supplemental Appendix Table 2
Selection of sources of evidence†	9	State the process for selecting sources of evidence (i.e., screening and eligibility) included in the scoping review.	#Study selection
Data charting process‡	10	Describe the methods of charting data from the included sources of evidence (e.g., calibrated forms or forms that have been tested by the team before their use, and	#Extraction and charting of the data

SECTION	ITEM	PRISMA-ScR CHECKLIST ITEM	REPORTED IN SECTION OR PAGE #
		whether data charting was done independently or in duplicate) and any processes for obtaining and confirming data from investigators.	
Data items	11	List and define all variables for which data were sought and any assumptions and simplifications made.	#Study Selection #Extraction and charting of the data
Critical appraisal of individual sources of evidence§	12	If done, provide a rationale for conducting a critical appraisal of included sources of evidence; describe the methods used and how this information was used in any data synthesis (if appropriate).	NA
Synthesis of results	13	Describe the methods of handling and summarizing the data that were charted.	#Collating, summarising and reporting results
**RESULTS**			
Selection of sources of evidence	14	Give numbers of sources of evidence screened, assessed for eligibility, and included in the review, with reasons for exclusions at each stage, ideally using a flow diagram.	#Results
Characteristics of sources of evidence	15	For each source of evidence, present characteristics for which data were charted and provide the citations.	#Study and Participant Characteristics
Critical appraisal within sources of evidence	16	If done, present data on critical appraisal of included sources of evidence (see item 12).	NA
Results of individual sources of evidence	17	For each included source of evidence, present the relevant data that were charted that relate to the review questions and objectives.	#Overview of auditory impairments following non-blast related TBI #Effect of severity of non-blast related TBI on auditory outcomes #Effect of aetiology of non-blast related TBI on auditory outcomes #Effect of gender on auditory outcomes following non-blast related TBI
Synthesis of results	18	Summarize and/or present the charting results as they relate to the review questions and objectives.	#Overview of auditory impairments following non-blast related TBI #Effect of severity of non-blast related TBI on auditory outcomes #Effect of aetiology of non-blast related

SECTION	ITEM	PRISMA-ScR CHECKLIST ITEM	REPORTED IN SECTION OR PAGE #
			TBI on auditory outcomes #Effect of gender on auditory outcomes following non-blast related TBI
**DISCUSSION**			
Summary of evidence	19	Summarize the main results (including an overview of concepts, themes, and types of evidence available), link to the review questions and objectives, and consider the relevance to key groups.	#Discussion
Limitations	20	Discuss the limitations of the scoping review process.	#Strengths and Limitations
Conclusions	21	Provide a general interpretation of the results with respect to the review questions and objectives, as well as potential implications and/or next steps.	#Conclusion
**FUNDING**			
Funding	22	Describe sources of funding for the included sources of evidence, as well as sources of funding for the scoping review. Describe the role of the funders of the scoping review.	#Funding

JBI = Joanna Briggs Institute; PRISMA-ScR = Preferred Reporting Items for Systematic reviews and Meta-Analyses extension for Scoping Reviews.
* Where *sources of evidence* (see second footnote) are compiled from, such as bibliographic databases, social media platforms, and Web sites.
† A more inclusive/heterogeneous term used to account for the different types of evidence or data sources (e.g., quantitative and/or qualitative research, expert opinion, and policy documents) that may be eligible in a scoping review as opposed to only studies. This is not to be confused with *information sources* (see first footnote).
‡ The frameworks by Arksey and O'Malley (6) and Levac and colleagues (7) and the JBI guidance (4, 5) refer to the process of data extraction in a scoping review as data charting.
§ The process of systematically examining research evidence to assess its validity, results, and relevance before using it to inform a decision. This term is used for items 12 and 19 instead of "risk of bias" (which is more applicable to systematic reviews of interventions) to include and acknowledge the various sources of evidence that may be used in a scoping review (e.g., quantitative and/or qualitative research, expert opinion, and policy document).

From: Tricco AC, Lillie E, Zarin W, O'Brien KK, Colquhoun H, Levac D, et al. PRISMA Extension for Scoping Reviews (PRISMAScR): Checklist and Explanation. Ann Intern Med. 2018;169:467–473. doi: 10.7326/M18-0850.

Table 2. Examples of database search strategy terms

Search terms	Search engine
Hearing OR Hyperacus* OR Hypoacus* OR Tinnitus OR Tympanomet*AND Traumatic Brain* OR Traumatic Subarachnoid OR ((H?emorrhage* OR H?ematoma* OR Bleed*) AND (Accident* OR Collision* OR Crash* OR Fall OR Falls OR Falling OR Athlet* OR Sport* OR Nonblast* OR "Non Blast" OR Cause OR Causes OR Causalit* OR Etiolog* OR Aetiolog*)	Medline via OvidSP
*Acoustic Impedance/ OR *Audiologist/ OR *Audiology/ OR *Evoked Response Audiometry/ OR *Pure Tone Audiometry/ OR exp *Speech Audiometry/ OR exp *Audiometry/ OR exp *Auditory Cortex/ OR * *Auditory Nervous System/ OR exp *Hearing/ OR exp *Auditory Threshold/ AND *Brain Concussion/ OR *Brain Hemorrhage/ OR *Diffuse Brain Injury/ OR *Brain Injury/ OR *Brain Ventricle Hemorrhage/ OR *Contrecoup Injury/ OR *Cranial Nerve Injury/ OR AND *Causality/ OR *Accident/ OR *Falling/ OR *Traffic *Sport Injury/ OR exp *Sport/ or exp *Violence/ OR Etiology.fs. OR (Accident* OR Collision* OR Crash* OR "Non Blast" OR Cause OR Causes OR Causalit* OR Etiolog* OR Aetiolog*	Embase
Deaf/ OR exp Ear Disorders/ OR Auditory Evoked Potentials/ OR exp Hearing Aids/ OR Loudness Perception/ OR Deaf/ OR Partially Hearing Impaired/ OR Pitch Discrimination/ OR Pitch Perception/ OR Acoustics/ OR Acoustic Reflex/ OR Auditory Localization/ OR Auditory Stimulation/ OR Speech Perception/ OR "Speech and Hearing Measures"/ OR exp Hearing Disorders/ OR Tinnitus/ AND Traumatic Brain Injury/ OR exp Head Injuries/ OR Brain Concussion/ OR	PsycINFO

Cerebral Hemorrhage/ OR exp Brain Injuries/ OR Subarachnoid Hemorrhage/ OR Axonotmes* OR Concussi* OR Brain Contusion* OR Brain Injur* OR Cerebellar Contusion* OR Cerebral Contusion* OR Cerebral Injur* OR Cerebral Trauma* OR Cerebrovascular Trauma* OR Contre-Coup Injur* OR Cortical Contusion* OR Cranial Nerve Injur* AND Home Accidents/ OR Industrial Accidents/ OR exp Sports/ OR exp Violence/ OR Collision* OR Crash* OR Fall OR Falls OR Falling OR Athlet* OR Sport* OR Nonblast* OR "Non Blast" OR Cause OR Causes OR Causalit* OR Etiolog* OR Aetiolog*	
Acoustic* OR Audio* OR Audition OR Auditory OR Autophon* OR Bone Conduction* OR Cochlear or Deaf* OR Dysacus* OR Electroacoust* OR Electrocochleogra* OR ENT OR Extracochlear AND Diffuse Axonal Injur* OR Forehead Trauma* OR Frontal Region Trauma* OR Head Injur* OR Head Trauma* OR Head Posttrauma* OR Nervous System Injur* OR Nervous System Trauma* OR Neurotmes* OR Occipital Region Trauma* OR Occipital Trauma* or Parietal Region Trauma* OR Postconcussi* OR Posttraumatic Brain* OR Posttraumatic Encephalopath* OR Posttraumatic AND "Non Blast" OR Cause OR Causes OR Causalit* OR Etiolog* or Aetiolog* OR Accident* or Collision* or Crash* or Fall or Falls or Falling or Athlet*	Science Citation Index Expanded
Hearing OR Hyperacus* OR Hypoacus* OR Listen* OR Loud* OR Neuroto* OR Noise* OR Olivocochlear OR Otorhin* OR Otic* OR Otoacoust* OR Otolaryng* OR Otolith* OR Otolog* OR Otoneuro* OR Otorhino* OR Otoscleros* OR Otoscop* OR Oto AND Forehead Trauma* OR Frontal Region Trauma* OR Head Injur* OR Head Trauma* OR Head Posttrauma* OR Nervous	SPORTDiscus

System Injur* OR Nervous System Trauma* OR Neurotmes* OR Occipital Region Trauma* OR Occipital Trauma* OR Parietal Region Trauma* OR Postconcussi* Posttraumatic Brain* OR Posttraumatic Encephalopath* OR Posttraumatic Subarachnoid OR TBI OR TBIs OR Temporal Region Trauma* OR Traumatic Brain* OR Traumatic Bulbar OR Traumatic Cereb* OR Traumatic Cranial	
Tinnitus OR Tonal OR Tone OR Tones OR Tympanomet* OR Acoustic* OR Audio* OR Audition OR Auditory OR Autophon* OR Bone Conduction* OR Cochlear OR Deaf* OR Dysacus* OR Electroacoust* OR Electrocochleogra* OR ENT OR Extracochlear AND Cranial OR Craniocerebralor Intracerebral OR Intracranial OR Medullary OR Midbrain OR Pontine OR Posterior Fossa OR Subarachnoid OR Subdural OR Skull Fracture* OR "Battle Sign" OR "Battle's Sign" OR "Battles Sign" OR Orbital Fracture* OR Blow-Out Fracture* OR Zygomatic Fracture* AND OR Collision* OR Crash* OR Fall OR Falls OR Falling OR Athlet* OR Sport* OR Nonblast* OR "Non Blast" OR Cause OR Causes OR Causalit* OR Etiolog* OR Aetiolog*	ProQuest Dissertations & Theses A&I

Table 3. A summary of auditory tests and PROMs

		**References (numbers)**
**Applied auditory tests**	Otoscopy, ENT/Otological/Neuro-otologic/Physical/Medical/Clinical examination	(11,22,23,30,35–37,39,44,46,51–53,55,56, 58,59,61–63,65,67,70–73,78)
	Pure-tone Audiometry	(11,22–43,45–67,69,71–73,75,76,78,80–82)
	Tympanometry	(11,34,36,37,40,43,46, 50,54,55,57,64,66,67,71–73,78)
	Acoustic Reflex Thresholds	(11,34,37,39,40,43,50,54, 57,67,71)
	Tuning Fork (Rinne and/or Weber)	(22,34,39,40,46,64–66,70)
	**Basic Speech audiometry**	(11,26,32,37,39,47,50,53, 56,57,63,66,71,73,78)
	Speech discrimination score	(26,32,50,57,66,71)
	Kana discrimination test	(41)
	Speech reception threshold	(11,37,53,73)
	Speech recognition threshold	(11,32,47,73)
	Word recognition in quiet	(11,73)
	**Advanced Speech Audiometry (Speech-in-Noise test)**	(11,47,73)
	Quick-SIN test	(11,73)
	Dichotic Digits Test	(47)
	1-,2-,3- pair Dichotic Digit Recognition performance	(11)
	Revised speech perception in noise test	(11)
	Words-in-Noise test	(73)
	Spatial release from masking Test	(73)
	**Site-of-lesion Tests**	(22,28,50,66)
	Tone decay test	(66)
	Békésy test	(50)
	Fowler (ABLB)	(22,28)
	**Otoacoustic Emissions (OAEs) and Suppression Test**	(37,47,50,66,71,72)
	TEOAE	(37,71,72)

	DPOAE	(37,47,50,66,71)
	OAE Suppression (medial olivocochlear suppression effect)	(72)
	**Electrophysiological Tests**	(34,36–38,40,41,46,47,50, 53,55,58,66,68,71,80)
	ECOG	(38)
	Auditory Brainstem Response	(34,36,37,40,41,46,47, 50,53,55,58,66,68,71,80)
	Middle Latency Response	(41,47)
	Late Latency Response	(66,71)
	Mismatch Negativity	(66)
	P300	(66)
	**Central Auditory Tests**	(11,33,41,47,73)
	Monoaural Temporal Fine Structure Perception	(73)
	Spectral ripple reversal detection	(73)
	Interaural phase difference task with 500 Hz stimuli	(73)
	Intearaural coherence (IC-2C2AFC task)	(73)
	The Duration Patterns Test	(47)
	The Frequency Patterns Test	(47)
	Time Compressed Speech	(47)
	Competing Sentences Test	(47)
	Dichotic Listening (non-forced attention, forced attention)	(33)
	Monoaural Testing	(33)
	Environmental Sound Identification Test	(41)
	Two-tone Discrimination Test	(41)
	Rhythm Pattern Discrimination	(41)
	Melody Recognition Test	(41)
	Sound Localization	(41)
	the Gaps-in-Noise test	(11)
	the 500-Hz masking level difference	(11)
**PROMS**	Speech, spatial and qualities of hearing scale	(73)

	Screening Checklist for Auditory Processing Adults	(71)
	Hearing Handicap Inventory for Adults	(11,71,74,82)
	Tinnitus Handicap Inventory	(71,74,82)
	Tinnitus Questionnaire	(57)
	Likert scale (0-5) for tinnitus amplitudes	(76)
	Hyperacusis Questionnaire	(11,74,82)
	Numeric Rating Scale for loudness, discomfort, annoyance, ignorability, and unpleasantness	(57)
	Post-Concussion Symptom Scale (PCSS)	(94)

Table 4. Auditory pathway regions assessed by auditory tests

Assessment method	Primary Auditory Region Assessed
Otoscopic assessment	External auditory canal, tympanic membrane, and the middle ear (77)
Pure Tone Audiometry	Air conduction: Sound travels through the external auditory canal, tympanic membrane, ossicles, and cochlea before reaching the cochlear nerve (a component of cranial nerve VIII) and continuing through the brainstem to the auditory cortex<br>Bone conduction: Sound waves are introduced directly to the cochlea through the vibration of a bone conduction oscillator on the mastoid process (79)
Site-of-Lesion Tests	Differentiates between cochlear and retrocochlear (auditory nerve) (83)
Tuning Fork Test (Weber and/or Rinne)	Air and bone conduction pathways; helps differentiate conductive versus sensorineural hearing loss (84, 85)
Tympanometry	Middle ear function (mobility of the tympanic membrane and ossicular chain) (86, 87)
Acoustic Reflex Thresholds	Auditory pathway integrity up to the superior olivary complex via stapedius muscle reflex (88)
Basic Speech Audiometry (e.g., SDS, SRT)	The ability to process speech in auditory centres, starting from the outer ear and ending with the auditory cortex (79)
Advanced Speech Tests (e.g., QuickSIN)	The ability to process speech in auditory centres, starting from the outer ear and ending with the cortex in noise (79)
OAEs (DPOAE, TEOAE)	Functionality of the outer hair cells in the cochlea (89)
OAE Suppression Test	Function of the efferent auditory system via the medial olivocochlear bundle (part of the central auditory nervous system) (90)
Electrophysiological Tests (e.g., ABR, MLR, LLR, P300, MMN)	Auditory pathway from the auditory nerve to more central regions in the brain, such as brainstem, thalamocortical pathways, auditory cortex (91)
Central Auditory Tests (e.g., dichotic listening, GIN, DDT)	Central auditory processing abilities (e.g., temporal processing, dichotic listening, auditory discrimination) (92)
